# Light‐Harvesting Protein LHCB4 Is Important for Stable Dimeric Organization of Photosystem II

**DOI:** 10.1111/ppl.71144

**Published:** 2026-10-09

**Authors:** Tereza Vánská, Monika Opatíková, Roman Kouřil, Zuzana Kučerová, Václav Karlický, Iva Ilíková, Marek Rác, Pavel Roudnický, Vojtěch Chýlek, Petr Ilík

**Affiliations:** ^1^ Department of Biophysics, Faculty of Science Palacký University Olomouc Czech Republic; ^2^ Department of Physics, Faculty of Science University of Ostrava Ostrava Czech Republic; ^3^ Global Change Research Institute of the Czech Academy of Sciences Brno Czech Republic; ^4^ Institute of Experimental Botany of the Czech Academy of Sciences, Centre of Plant Structural and Functional Genomics Olomouc Czech Republic; ^5^ Central European Institute of Technology Masaryk University Brno Czech Republic

## Abstract

Light‐harvesting protein LHCB4 is a monomeric antenna of photosystem II (PSII) that connects the PSII core (C) with strongly (S) and moderately (M) bound LHCII trimers, forming the stable dimeric supercomplex C_2_S_2_M_2_. LHCB4 is known to stabilize LHCII attachment to the PSII core dimer (C_2_). Here, we show that LHCB4 is essential for the intrinsic stability of the PSII core dimer itself, a fundamental structural unit required for efficient photochemistry. In the *lhcb4* mutant, clear‐native PAGE revealed the absence of C_2_S_2_M_(2)_ and only small amounts of C_2_S_2_ and C_2_S supercomplexes. Single‐particle electron microscopy identified two types of C_2_S_(2)_ supercomplexes: a canonical configuration with an empty LHCB4 site and a more abundant “packed” structure, arising from a relative shift of the C(S) monomers. However, most PSII particles were present as monomeric CS complexes with attached LHCB5 antenna. In contrast, PSII analyzed directly in thylakoid membranes retained the canonical C_2_ organization and even formed nonparallel associations of C_2_S_2_ supercomplexes, indicating that the “packed” C_2_S_2_ and CS forms arise during isolation due to reduced PSII dimer stability. The PSII core dimer also readily disintegrates in the mutant lacking all monomeric antenna proteins (NoM), implying that LHCB4 is critical for the stability of the C_2_ dimer itself. Consistently, *lhcb4* plants exhibited reduced thermal stability of PSII macro‐organization and photochemistry in vivo. Our findings demonstrate that LHCB4 is not merely a peripheral antenna connector, but also an important structural determinant of PSII core dimer stability, underscoring its essential role in efficient photochemistry and photoprotection.

## 1 Introduction

The primary reactions of oxygenic photosynthesis involve balanced cooperation of two supramolecular pigment–protein complexes embedded in the thylakoid membrane—photosystem I (PSI) and photosystem II (PSII). Both photosystems consist of a core complex and associated light‐harvesting complexes (LHCs), which increase the capacity for light capture. The absorbed light energy is transferred to the reaction centers in the photosystem cores, where it drives photochemical reactions.

From a structural point of view, a fully assembled PSII consists of a dimeric core complex (C_2_), an outer layer of minor LHCs, and the major light‐harvesting complex II (LHCII). The minor LHCs, represented by LHCB4, LHCB5, and LHCB6 antenna proteins (also known as CP29, CP26, and CP24, respectively), are monomeric, whereas LHCIIs are present as trimers composed of various combinations of the LHCB1, LHCB2, and LHCB3 proteins (see e.g., Caffarri et al. 2009; Croce and van Amerongen 2011; Xu et al. 2017). In land plants, the LHCII antenna typically comprises two strongly bound trimers (S_2_), formed by a combination of LHCB1 and LHCB2 (Hankamer et al. 1997) or by LHCB1 only (in spruce, Opatíková et al. 2023), and two moderately bound trimers (M_2_, formed by LHCB1–3) (see Croce and van Amerongen 2011). The trimeric LHCIIs are connected to the PSII core dimer (C_2_) by all three minor antennae LHCB4–6, forming the stable supercomplex C_2_S_2_M_2_ (Albanese et al. 2016; Caffarri et al. 2009; Su et al. 2017). The only known exception to date is the *Pinaceae* family, where the antenna proteins LHCB3 and LHCB6 are absent (Kouřil et al. 2016, 2020; Opatíková et al. 2023), and LHCB4 is constitutively replaced by its homolog LHCB8 (formerly called LHCB4.3, Grebe et al. 2019).

From a functional perspective, PSII supercomplexes with large antennae, such as C_2_S_2_M_2_, are essential for efficient photosynthesis under low light. When plants acclimate from low‐ to high‐light conditions, this configuration typically shifts from C_2_S_2_M_2_ to C_2_S_2_, the smallest physiologically relevant PSII supercomplex (Albanese et al. 2016; Ballottari et al. 2007; Kouřil et al. 2013; Morosinotto et al. 2006). This transition is accompanied by downregulation of LHCB3 and LHCB6 (Kouřil et al. 2013; Štroch et al. 2022) and upregulation of LHCB8 (Floris et al. 2013). Since LHCB8 has reduced affinity for LHCB6 (Albanese et al. 2016, 2018, 2019), the replacement of LHCB4 with LHCB8 likely plays a key role in reducing PSII antenna size during high‐light acclimation. A recent study by Caferri et al. (2025) demonstrated that constitutive replacement of LHCB4 with LHCB8 in an *Arabidopsis* mutant prevents C_2_S_2_M_2_ assembly, leaving PSII supercomplexes in the C_2_S_2_ state.

The facts outlined above imply that the minor antenna proteins LHCB4/LHCB8 play an important role in the organization of PSII. LHCB4/LHCB8 are encoded by three genes *LHCB4.1*, *LHCB4.2* (for LHCB4.1 and LHCB4.2 proteins), and *LHCB4.3* (for LHCB8). Under normal conditions, the first two are expressed at comparable levels, whereas *LHCB4.3* expression is roughly 20 times lower, placing LHCB8 among the rare LHC proteins (Ilíková et al. 2021; Jansson 1999; Klimmek et al. 2006; Zybailov et al. 2008). For simplicity, we will use the designation LHCB4 for all proteins LHCB4.1, LHCB4.2, and LHCB8.

The importance of LHCB4 for PSII supercomplex assembly and function has been studied in Arabidopsis using antisense *aslhcb4* plants (van Oort et al. 2010; Yakushevska et al. 2003) as well as insertion triple mutant *lhcb4* (*lhcb4.1 lhcb4.2 lhcb4.3*) (Betterle et al. 2009; de Bianchi et al. 2011; Chen et al. 2018; Miloslavina et al. 2011). Using nondenaturing Deriphat‐PAGE, it has been shown that the absence of all LHCB4 isoforms destabilizes the attachment of LHCII trimers to the PSII core dimer, which leads to a significant decrease in PSII supercomplex level (de Bianchi et al. 2011; Caferri et al. 2025; Guardini et al. 2022). Nevertheless, analysis of a small number of PSII particles from the *lhcb4* mutant using single particle electron microscopy (EM) suggested the presence of C_2_S_2_ supercomplexes with an abnormal shape, although a detailed structural description of this form of the PSII supercomplex was limited by low resolution (de Bianchi et al. 2011). In addition to these structural effects, the absence of LHCB4 was reported to increase susceptibility of PSII to photoinhibition (de Bianchi et al. 2011) and to lower the extent of nonphotochemical quenching (NPQ amplitude, e.g., de Bianchi et al. 2011), indicating an important role of LHCB4 in the NPQ process (Dall'Osto et al. 2017; Lapillo et al. 2020; Nicol et al. 2019).

In this study, we investigated PSII assemblies in Arabidopsis lhcb4 mutant lacking all LHCB4 isoforms using a combination of very mild clear‐native PAGE (CN‐PAGE) and single‐particle EM of separated PSII (super)complexes. This approach, validated in our previous studies on the structural roles of the other two monomeric LHCB proteins, LHCB6 (Ilíková et al. 2021) and LHCB5 (Vánská et al. 2025), documents an additional role of LHCB4. The absence of this minor antenna not only destabilizes M‐trimer tethering to the PSII core but also weakens the interaction between PSII core monomers within the C_2_ dimer. We also showed that the structural modification of PSII assemblies due to the absence of LHCB4 destabilizes PSII in vivo, which likely underlies the higher stress sensitivity of the *lhcb4* mutant.

## 2 Materials and Methods

### 2.1 Plant Material

*Arabidopsis thaliana* wild type (WT, accession Columbia) and the triple *lhcb4* mutant (*lhcb4.1 lhcb4.2 lhcb4.3*, de Bianchi et al. 2011) were grown in soil in a FytoScope Walk‐In chamber (Photon Systems Instruments, Drásov, Czech Republic) for 6 weeks. The growth conditions in the chamber were as follows: 8/16 h light/dark photoperiod, 22/20°C day/night temperature, 110 μmol photons m^−2^ s^−1^ (400–700 nm), and 60% relative humidity.

### 2.2 Isolation of Thylakoid Membranes

*Arabidopsis* plants were dark‐adapted for 30 min, after which the rosettes were cut at the base and immediately processed for thylakoid isolation, following the protocol of Dau et al. (1995) with modifications. Plant material was homogenized in ice‐cold grinding buffer containing 35 mM HEPES/NaOH (pH 7.2), 0.4 M NaCl, 0.4 M sucrose, 4 mM MgCl_2_·6H_2_O, 5 mM sodium ascorbate, and 2 mg mL^−1^ BSA. The homogenate was filtered through a double layer of Miracloth and centrifuged at 5000 *g* for 6 min at 4°C. The pellet was resuspended in resuspension buffer (25 mM HEPES/NaOH, pH 7.5, 150 mM NaCl, 8 mM MgCl_2_·6H_2_O, 0.9 mM EDTA) and centrifuged again under the same conditions for 10 min. For the final wash, the pellet was resuspended in storage buffer (50 mM HEPES/NaOH, pH 7.2, 0.4 M sucrose, 15 mM NaCl, 5 mM MgCl_2_·6H_2_O) and briefly centrifuged (5000 *g*, 5 min, 4°C). The resulting pellet, containing thylakoid membranes, was gently resuspended in a minimal volume of storage buffer. All steps were carried out at 4°C or on ice under dim green light.

### 2.3 Clear Native Polyacrylamide Gel Electrophoresis (CN‐PAGE)

CN‐PAGE was performed according to Nosek et al. (2017), with minor modifications as described by Vánská et al. (2025). Thylakoid membrane suspensions were solubilized using n‐dodecyl‐α‐d‐maltopyranoside (α‐DDM) at a detergent‐to‐chlorophyll mass ratio of 10:1 for subsequent single‐particle analysis (Figure 3) and 5:1 for subsequent Western blot analysis (Figure 5). The thylakoid–detergent mixture was supplemented with sample buffer (50 mM HEPES/NaOH, pH 7.2, 0.4 M sucrose, 5 mM MgCl_2_, 15 mM NaCl, and 10% glycerol), bringing the total volume to 30 μL. After gentle mixing, the samples were incubated for 1 min at 4°C and centrifuged at 20,000 *g* for 10 min at 4°C. The resulting supernatants were loaded onto 4%–8% polyacrylamide gradient gels (10 μg of chlorophyll per lane) prepared as described by Wittig et al. (2007) except that no stacking gel was used and the samples were loaded directly onto the resolving gel.

Electrophoresis was performed using the Bio‐Rad Mini‐PROTEAN Tetra Cell system with a Bis–Tris/Tricine buffer system: cathode buffer (50 mM Tricine, 15 mM Bis–Tris/HCl, pH 7.0) and anode buffer (50 mM Bis–Tris/HCl, pH 7.0). Gels were run under cooled conditions in the dark. The initial separation was conducted at a constant current of 3.5 mA per gel for 15 min, followed by a step increase to 7 mA maintained until the migration front of chlorophyll pigments reached the bottom edge of the gel. Visualization of native protein complexes was performed using an Amersham Imager 600RGB system (GE Healthcare Life Sciences) in transmission mode under white light illumination and in fluorescence mode with an excitation wavelength of 460 nm and a bandpass filter of 690–720 nm.

### 2.4 2D‐PAGE and Immunoblotting

2D‐SDS‐PAGE was performed according to Laemmli (1970) modified according to Opatíková and Kouřil (2024). The selected sections of the CN‐PAGE gel strips from the first dimension were incubated in SDS buffer (25 mM Tris (pH 7.5/HCl), 35 mM SDS, 15 mM DTT) for 35 min and then applied to the SDS gel. The electrophoresis conditions were as follows: 5 mA/30 min = > 10 mA/2 h = > 20 mA/1.5 h at room temperature.

The separated proteins were transferred from the SDS gel to a polyvinylidene fluoride membrane using the Trans‐Blot Turbo RTA Mini 0.2 lm PVDF Transfer Kit (BioRad) with conditions 2.5 A, 25 V for 25 min. The presence of the PSBB (CP47) protein was detected using a primary Anti‐PSBB antibody (AS04 038, Agrisera) in tandem combination with the secondary antibody with conjugated horseradish peroxidase (HRP) enzyme. The chemiluminescent signal was recorded after developing with Immobilon Western Chemiluminescent HRP Substrate (Merck) and visualized using a gel scanner Amersham Imager 600RGB (GE HealthCare Life Sciences).

### 2.5 Electron Microscopy and Single Particle Analysis

The selected CN‐PAGE bands were subjected to single‐particle electron microscopy analysis. The gel bands were excised, finely chopped, and incubated in elution buffer (50 mM HEPES (pH 7.2), 15 mM NaCl, 5 mM MgCl_2_, and α‐DDM at its critical micellar concentration 0.008%). Gel pieces were submerged in the elution buffer and incubated overnight at 4°C to allow passive elution of the pigment‐protein complexes. The resulting supernatants were used directly for subsequent specimen preparation for transmission electron microscopy without further processing according to Kouřil et al. (2014) by applying them to glow‐discharged carbon‐coated copper grids followed by negative staining with 2% uranyl acetate.

Electron micrographs were collected using a Tecnai G2 F20 microscope (FEI Technologies) with an Eagle 4 K CCD camera (FEI Technologies, Hillsboro, USA) at ×134028 magnification. The pixel size at the specimen level after binning the images to 2048 × 2048 pixels was 0.224 nm. The 160,100, 45,618, 27,495, 64,421, and 64,374 projections were picked in semiautomated mode from 5152, 3664, 8549, 4168, and 4773 micrographs of specimens prepared from the gel bands assigned as the PSI/C_2_S/CS, C_2_S, C_2_S_2_ (*lhcb4*), PSI/C_2_S/CS (WT), and PSI/C_2_S/CS (NoM), respectively. The data sets were subjected to reference‐free 2D classification using the SCIPION image processing framework (de la Rosa‐Trevín et al. 2016). In the *lhcb4* mutant, the C_2_S_2_ band contained overall ~7883 and ~2169 particles assigned to “packed” and canonical C_2_S_2_ form, respectively; the C_2_S band contained overall ~5033 and ~3589 particles assigned to “packed” and canonical C_2_S form, respectively; the PSI/CS/C_2_ band contained overall ~66,226 and ~3888 particles assigned to CS and C_2_ PSII form, respectively. For WT, the PSI/C_2_/CS band contained overall ~3075 and ~3822 particles assigned to CS and C_2_ PSII form, respectively.

Electron micrographs of grana membranes from *A. thaliana* triple *lhcb4* mutant (209 micrographs), isolated according to Kouřil et al. (2013), were recorded at ×83562 magnification. The pixel size at the specimen level after binning the images to 2048 × 2048 pixels was 0.36 nm. Analysis focused on the identification of specific PSII megacomplexes in the grana membranes was performed using the SCIPION framework, where approximately 97,000 projections of PSII particles were initially selected in manual and auto‐picking modes (de la Rosa‐Trevín et al. 2013; Scheres 2015), which were analyzed and further classified into specific classes. To determine the minimal distances between PSII complexes in the grana membranes from *A. thaliana* triple *lhcb4* mutant, 29,155 PSII projections were selected using SCIPION from micrographs. The coordinates of the PSII particles were then exported and analyzed using Microsoft Excel.

### 2.6 Mass Spectrometry

Proteins extracted from thylakoid membranes were subjected to filter‐aided sample preparation as described elsewhere (Wiśniewski et al. 2009). Peptide solutions were analyzed by liquid chromatography–tandem mass spectrometry (LC–MS/MS), performed using an UltiMate 3000 RSLCnano system (Thermo Fisher Scientific) online‐coupled with an Orbitrap Exploris 480 mass spectrometer (Thermo Fisher Scientific). LC–MS/MS data, acquired in data‐dependent acquisition (DDA) mode, were processed using MaxQuant, and results tables were evaluated in the KNIME Analytics Platform within a containerized environment (https://github.com/OmicsWorkflows/KNIME_docker_vnc). See the Supporting Information for full details regarding the analyses and data evaluation.

### 2.7 Chlorophyll Fluorescence and P700 Absorption Measurements

Measurements were performed on attached, fully expanded leaves. Plants were sampled at mid‐photoperiod and dark‐adapted for 30 min prior to measurements. All measurements were performed under dim green light. Very fast chlorophyll fluorescence induction curves (OJIP) were recorded using a Plant Efficiency Analyser (PEA; Hansatech) on the adaxial side of dark‐adapted leaves. A 10‐s saturating red‐light pulse (6600 μmol photons m^−2^ s^−1^) was applied. The maximum quantum yield of PSII photochemistry was calculated as *F*_V_/*F*_M_ (*F*_V_ = *F*_M_ − *F*_O_). Fluorescence parameters were evaluated using Biolyzer software (R. M. Rodríguez, University of Geneva). Simultaneous PSI and PSII performance was assessed using a Dual‐PAM‐100 system (Heinz Walz). Measurements were carried out under red actinic light (800 μmol photons m^−2^ s^−1^) combined with saturating pulses (10,000 μmol photons m^−2^ s^−1^, 300 ms). Calculated parameters were obtained using Dual‐PAM software (Klughammer and Schreiber 2008a, 2008b, see also Kramer et al. 2004). To obtain the signal corresponding to the fully oxidized primary donor of PSI (P700), an automated Dual‐PAM routine was performed on dark‐adapted leaves before illumination with actinic light. The routine involved a preprogrammed saturation pulse applied after 10 s of far‐red preillumination. The F_O_^' ^values required for the calculation of photosystem II parameters during the induction measurement were calculated using the approximation of Oxborough and Baker (1997), as described in the manual for the Dual‐PAM‐100 device.

### 2.8 Circular Dichroism (CD) Spectroscopy

Room temperature CD spectra were recorded in the range of 400–750 nm with a J‐815 spectropolarimeter (Jasco). Intact leaves were supported by a flat cell, and CD spectra were measured with a measuring beam perpendicular to the leaf similarly (Tóth et al. 2016). The spectra of intact leaves were recorded in steps of 0.5 nm with an integration time of 1 s, a band‐pass of 3 nm, and a scanning speed of 100 nm min^−1^. For temperature dependences of CD spectra, the samples were thermostated in a Peltier folder (Jasco) in the temperature range of 20°C–70°C in 2°C increments with 1.5 min incubation at each measuring temperature before recording the spectra. The CD spectra were normalized to the chlorophyll Q_Y_ absorption band. In order to minimize the influences of the overlapping excitonic CD bands, the amplitudes of the (+)685 nm and (+)505 nm ψ‐type CD bands were calculated as the difference of the CD signal at (+)685 nm to 730 nm and (+)505 to 550 nm. Measured temperature dependences of the CD bands were fitted with a sigmoidal curve that resulted in the estimation of transition temperatures (Tm). Tm marks the disassembly of the chiral macrodomains of the complexes and is defined as the temperature at which the intensity of the CD band is decreased to 50% of its value at 20°C.

### 2.9 Fluorescence Temperature Curves (FTC)

Thermostability of PSII function in the WT and *lhcb4* mutant was assessed using a PlanTherm PT100 instrument (Photon Systems Instruments, Drásov, Czech Republic) as described by Ilík et al. (2018). Prior to the measurements, plants were dark‐adapted for 30 min, after which the leaves were detached. Samples were heated from room temperature to 70°C in a water bath at a rate of 2°C min^−1^. Simultaneously, chlorophyll fluorescence intensity (F_O_ level, minimal fluorescence) was recorded and plotted as a function of temperature (FTC). The temperature corresponding to the maximum curvature point of the FTC during the fluorescence rise was calculated using ProfileCon software (Photon Systems Instruments).

### 2.10 Gas Exchange Measurements

Net CO_2_ assimilation rate (A) was determined using a GFS‐3000 portable gas exchange system (Heinz Walz GmbH). The adaxial side of the measured leaf was illuminated with a mixture of red (90%) and blue (10%) light at an irradiance of 95 μmol photons m^−2^ s^−1^ (PAR). Airflow through the leaf chamber was maintained at 400 μmol s^−1^ with the chamber temperature at 23°C. Concentrations of CO_2_ and H_2_O were set as 400 μmol mol^−1^ and 17,100 μmol mol^−1^, respectively. This setup ensured 60% relative humidity in the chamber. These conditions were selected to mimic the conditions in the growth chamber. For the measurements, we used fully expanded leaves of 6‐week‐old plants from the growth chamber without dark acclimation. The data points were recorded after stabilization (2–3 min). The net CO_2_ assimilation rate (A) (μmol CO_2_ m^−2^ s^−1^) was calculated according to von Caemmerer and Farquhar (1981).

### 2.11 Thermoluminescence Measurements

Thermoluminescence curves were measured using a Thermoluminescence TL 6000/ST device (Photon Systems Instruments). Thylakoid membranes (see above) were resuspended in buffer (50 mM HEPES, pH 7.2, 5 mM MgCl_2_, 15 mM NaCl) with or without DCMU (5 μM). Four hundred microliters of the thylakoid membrane suspension corresponding to 18 μg of chlorophylls was placed on a metallic disc and cooled from room temperature to 5°C, followed by one single turnover flash prior to the measurement. The thermoluminescence curves were recorded while the sample was warmed up to 85°C at a heating rate of 0.5°C s^−1^.

## 3 Results and Discussion

### 3.1 Phenotypic Characterization and PSII Function of the *lhcb4* Mutant

The Arabidopsis *lhcb4* mutant has previously been studied and many valuable insights into the structural and physiological roles of LHCB4 have been obtained (Betterle et al. 2009; de Bianchi et al. 2011; Chen et al. 2018; Miloslavina et al. 2011). However, plant phenotypes can vary significantly even with minor changes in growth conditions (e.g., compare *lhcb6* mutant phenotypes in Ilíková et al. 2021, and de Bianchi et al. 2008). Therefore, as the first step, we performed comprehensive characterization of the *lhcb4* mutant under our growth conditions to verify that its key phenotypic characteristics align with previously published data, ensuring valid comparisons with earlier studies.

The *lhcb4* mutant showed no clear phenotypic differences compared to WT (Figure 1A) and the fresh weight of rosettes as well as pigment concentrations of the mutant and WT were comparable (Table S1, see also de Bianchi et al. 2011). The absence of LHCB4 proteins in the *lhcb4* plants was verified by immunoblotting (Figure 1B) and by mass spectrometry (MS) analysis of isolated thylakoid membranes (Figure 1C). The latter also confirmed that the *lhcb4* mutant has lower content of LHCB3 (see also Chen et al. 2018) and a pronounced reduction in the relative amount of LHCB6 (Figure 1C; see also Andersson et al. 2001; de Bianchi et al. 2011). Our proteomic analysis additionally showed that no other LHCB protein is significantly affected and that the loss of LHCB4 does not markedly affect relative abundances of the major photosynthetic complexes (PSI, PSII, LHCII, cytochrome b_6_/f complex, and ATP synthase; Figure 1D).

**FIGURE 1:**
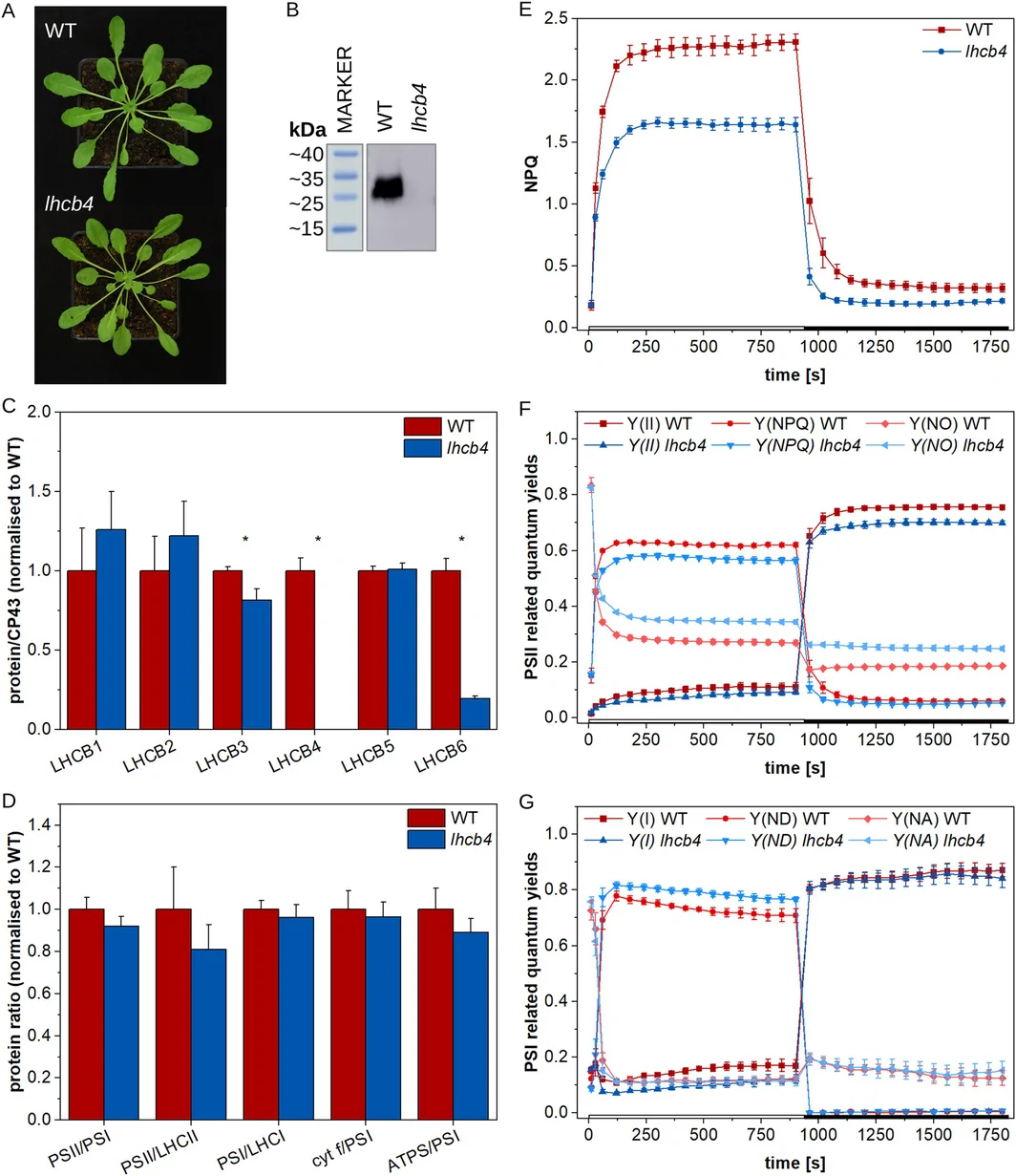
Phenotype and photosynthetic characteristics of the *lhcb4* mutant. (A) Phenotype of *Arabidopsis thaliana* wild type (WT) and *lhcb4* mutant plants grown at controlled conditions for 6 weeks (8/16 h light/dark cycle; 22/20°C; 110 μmol photons m^−2^ s^−1^; 60% humidity). (B) Immunoblot analysis of thylakoid membranes of WT and *lhcb4* mutant plants with antibody directed against LHCB4. (C) Content of light‐harvesting proteins LHCB1‐6 evaluated relatively to the content of CP43 protein in the WT and the *lhcb4* mutant. The protein content was determined in isolated thylakoid membranes by liquid chromatography–tandem mass spectrometry (LC–MS/MS). The columns represent means + SD (*n* = 3). (D) Protein ratios of photosynthesis‐related thylakoid membrane proteins of the WT and the *lhcb4* mutant. The protein content was determined by LC–MS/MS in isolated thylakoid membranes. PSII represents the sum of relative PG intensities of D1, D2, CP43, and CP47 proteins, LHCII—LHCB1–3 proteins, PSI—PSAA and PSAB proteins, LHCI—LHCA1–4 proteins, ATPS—α and β subunits of ATP synthase, and cyt f represents cytochrome f component of cytochrome b_6_f complex. The columns represent means + SD (*n* = 3). (E–G) Parameters of photosystem I and II photosynthetic activity during light acclimation and subsequent dark relaxation in WT and *lhcb4* mutant plants. Measurements were performed during actinic light exposure (indicated by white bar, 800 μmol photons m^−2^ s^−1^) followed by dark relaxation (indicated by black bar), using saturating light pulses (300 ms, 10,000 μmol photons m^−2^ s^−1^). Data represent mean values ± SD (*n* = 5). Plants were dark‐adapted for 30 min prior to measurements. (E) Nonphotochemical quenching (NPQ). (F) Quantum yield of PSII photochemistry—Y(II), quantum yield of light‐regulated nonphotochemical quenching—Y(NPQ), and quantum yield of nonregulated nonphotochemical energy dissipation—Y(NO). (G) Quantum yield of PSI photochemistry—Y(I), quantum yield of nonphotochemical energy dissipation due to donor‐side limitation—Y(ND), and quantum yield of nonphotochemical energy dissipation due to acceptor‐side limitation—Y(NA). Data in panels (C) and (D) were tested for statistical significance. Datasets passing normality and equal variance tests were analyzed using Student's *t*‐test, whereas datasets failing these assumptions were evaluated using Mann–Whitney rank sum test. Statistically significant differences are indicated by an asterisk (*α* ≤ 0.05).

To evaluate the basic functional photosynthetic parameters of the *lhcb4* mutant, we performed the measurements of chlorophyll fluorescence induction on leaves. The maximum quantum yield of PSII photochemistry (F_V_/F_M_ ratio) was lower in the *lhcb4* mutants compared to WT, which is a result of significantly higher minimum chlorophyll fluorescence level F_O_ (Table S1, Andersson et al. 2001; de Bianchi et al. 2011). The increase in F_O_ is often interpreted as the presence of light‐harvesting complexes loosely bound to the PSII core subunits (e.g., Miloslavina et al. 2011; Ware et al. 2015), although the functional antenna size of PSII (TR_0_/RC parameter, Table S1) was similar in both *lhcb4* mutant and in WT (see also, e.g., Miloslavina et al. 2011). The CO_2_ assimilation rate of *lhcb4* plants measured under growth conditions was also not significantly different from that of WT, which corresponds with similar biomass of both plant genotypes (Table S1).

Analysis of chlorophyll fluorescence parameters during slow fluorescence induction under high light conditions (800 μmol photons m^−2^ s^−1^) revealed a slightly lower yield of PSII photochemistry (Y(II)) in the *lhcb4* plants (Figure 1F), which coincides with a higher donor‐side limitation of PSI (Y(ND)) and with a lower quantum yield of PSI photochemistry (Y(I)) (Figure 1G). Our data confirm the findings of previous studies that *lhcb4* plants have lower nonphotochemical quenching parameter NPQ than WT (Figure 1E, Andersson et al. 2001; Betterle et al. 2009; de Bianchi et al. 2011; Guardini et al. 2020). The NPQ parameter can be expressed as a ratio between regulated (Y(NPQ)) and nonregulated (Y(NO)) nonphotochemical quenching (Klughammer and Schreiber 2008a). Thus, the lower NPQ reflects higher Y(NO) relative to Y(NPQ) (see Figure 1F), suggesting a shift from regulated toward nonregulated energy dissipation in the *lhcb4* mutant. This altered energy partitioning could be associated with the previously reported higher susceptibility of the *lhcb4* mutant to photodamage (de Bianchi et al. 2011).

From a structural perspective, the elevated Y(NO) may arise from compromised architectural or functional integrity of the PSII complex, which could affect electron transfer and recombination pathways within the PSII core. To evaluate this possibility, we used PSII thermoluminescence to sensitively probe the energetic states of PSII redox cofactors in isolated thylakoids (for a review see, e.g., Ducruet and Vass 2009). The thermoluminescence B band, reflecting the recombination of the Q_B_^−^S_2(3)_^+^ charge pair, was slightly decreased and shifted to higher temperatures when compared to WT (Figure 2), suggesting increased stability of this charge pair. In contrast, the peak temperature of the thermoluminescence Q band (detected with an inhibitor of the electron transfer between Q_A_ and Q_B_), reflecting the recombination of the Q_A_^−^S_2_^+^ charge pair, was similar in *lhcb4* and WT, whereas its amplitude was markedly reduced in the mutant (Figure 2). Given that overall PSII content and functional antenna size are comparable between genotypes (see Figure 1D and TR_0_/RC in Table S1), the lower Q‐band amplitude could reflect a higher P680^+^Pheo^−^ and/or P680^+^Q_A_^−^ recombinations in the PSII core of the *lhcb4* mutant. Higher probability of these recombinations can explain the higher level of Y(NO) (see e.g., Barbato et al. 2020) in the *lhcb4* mutant (Figure 1F). The higher P680^+^Pheo^−^ recombination promotes the formation of P680 in its triplet state, leading to the singlet oxygen generation and enhanced photooxidative damage (for a review, see Vass 2012). This mechanism may also explain the previously observed higher susceptibility of PSII to photoinhibition in the *lhcb4* mutant (de Bianchi et al. 2011).

**FIGURE 2:**
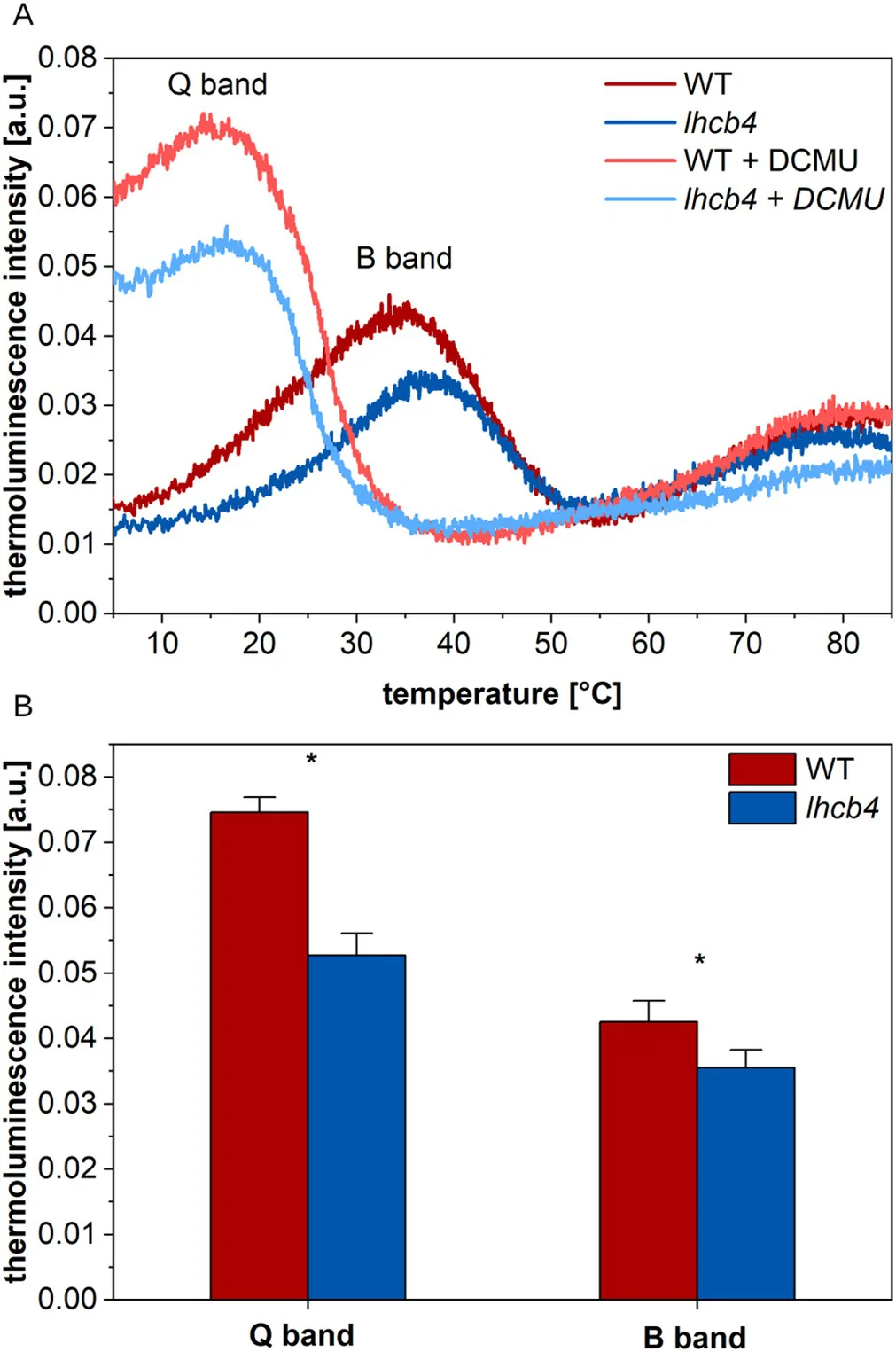
Chlorophyll thermoluminescence in thylakoid membranes isolated from wild type (WT) and the *lhcb4* mutant. (A) Representative chlorophyll thermoluminescence curves for WT and *lhcb4* mutant. Thylakoid membranes were untreated (to obtain the thermoluminescence B band, recombination of Q_B_^−^S_2(3)_^+^) or treated with 5 μM DCMU (to obtain the Q band, recombination of Q_A_^−^S_2_^+^). Suspensions of thylakoid membranes were cooled from room temperature to 5°C, followed by one single‐turnover flash. Thermoluminescence curves were recorded while the samples were warmed up. (B) Amplitudes of the B and Q bands for WT and *lhcb4* mutant. Statistically significant differences are indicated by an asterisk (*α* ≤ 0.05). Number of repetitions was 3–9.

Although the *lhcb4* mutant shows normal growth, pigment content, and photosynthetic capacity, chlorophyll fluorescence and thermoluminescence analyses reveal subtle but significant PSII alterations: elevated Y(NO), stabilized Q_B_^−^S_2(3)_^+^ pair, and possible higher charge recombinations within PSII, likely increasing photodamage susceptibility. These functional changes suggest that the loss of LHCB4 likely leads to some perturbations in PSII organization, providing a strong rationale for a structural analysis of PSII supercomplexes to link these functional effects to specific architectural rearrangements.

### 3.2 PSII Supercomplexes in the *lhcb4* Mutant

Mild separation of photosynthetic complexes by CN‐PAGE (Figure 3A) confirmed that *lhcb4* mutants have significantly lower amount of PSII supercomplexes than WT, a result which agrees with previous studies (de Bianchi et al. 2011; Caferri et al. 2025; Guardini et al. 2022). Figure 3A shows both visible‐light image and a black‐and‐white fluorescent scan of separated pigment–protein complexes from WT and *lhcb4* thylakoid membranes. In the WT sample, a CN‐PAGE band pattern typical for *Arabidopsis thaliana* is observed (see, e.g., Nosek et al. 2017; Ilíková et al. 2021; Vánská et al. 2025). It shows large PSII assemblies (megacomplexes; PSII mc), a set of bands with PSII supercomplexes with different numbers of LHCII trimers (C_2_S_2_M_2_, C_2_S_2_M, C_2_S_2_/C_2_SM, C_2_S), distinct bands corresponding to PSI with co‐migrating PSII core dimer C_2_ (PSI/C_2_) and free light‐harvesting complexes (LHCs). We denoted the PSI/C_2_ band as PSI/C_2_/CS due to the appearance of CS complexes (see below).

**FIGURE 3:**
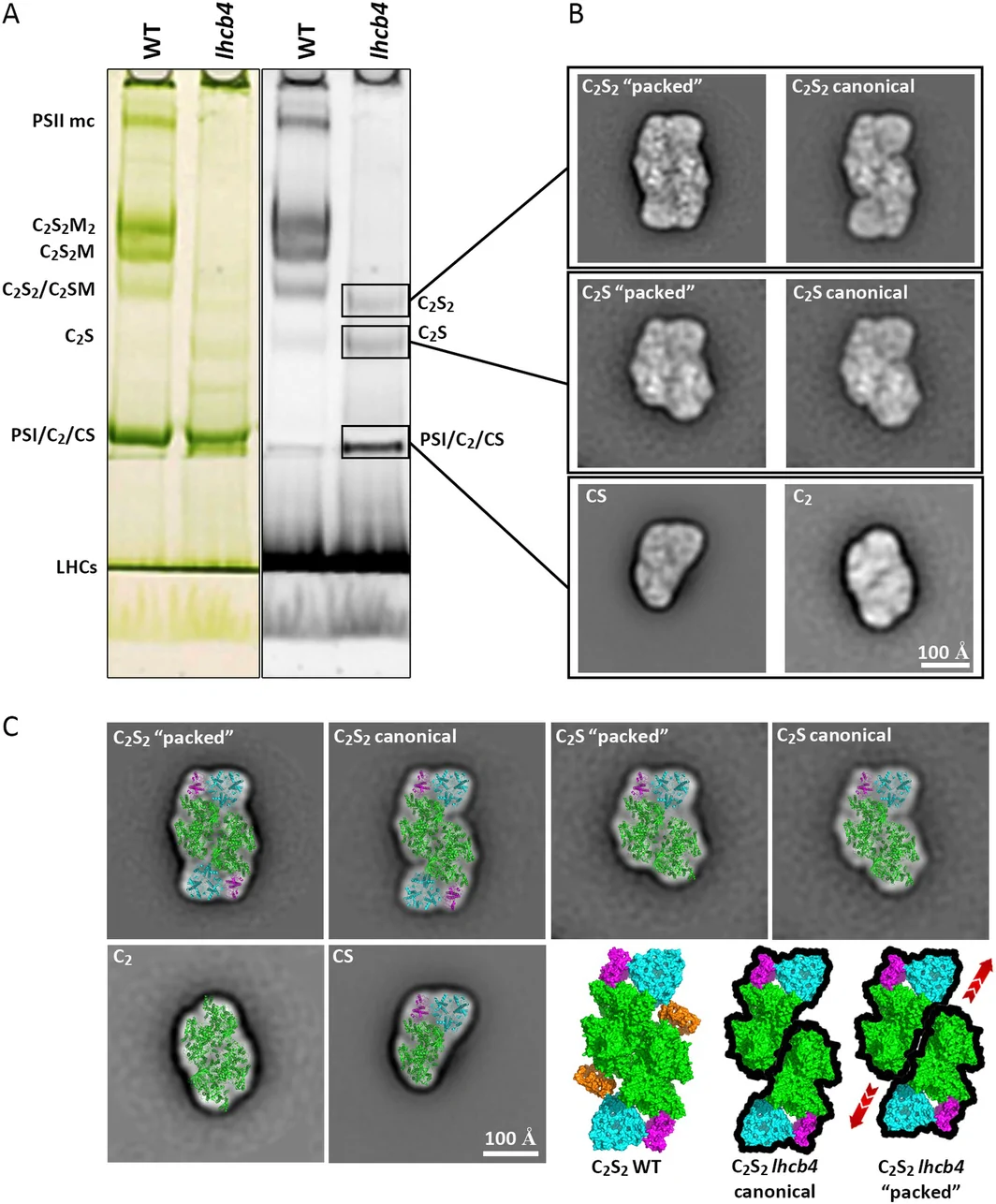
Separation and structural characterization of PSII assemblies from *lhcb4* mutant plants. (A) Separation of pigment–protein complexes from thylakoid membranes of WT and *lhcb4* mutant plants by CN‐PAGE. Thylakoid membranes were solubilized by 0.33% *n*‐dodecyl α‐d‐maltopyranoside (α‐DDM). The image shows gel lines scanned at room temperature in transmission mode (color image) and in fluorescence mode (black and white image). (B) Electron density maps of the two most abundant forms of PSII (super‐)complexes from *lhcb4* mutant in selected gel bands revealed by single particle electron microscopy (lumenal view). For projection maps of separated particles, see Figures S1–S3. (C) Fitting of the projection maps in (B) by truncated, high‐resolution model of PSII supercomplex (PDB: 5MDX) and a comparison of the fitted C_2_S_2_ structural models (surface models) from *lhcb4* and the C_2_S_2_ from WT. Black outlines highlight the contours of the individual CS monomers, and red arrows indicate their relative shift in the “packed” C_2_S_2_ structure. A direct visual comparison of the canonical and “packed” C_2_S_2_ is provided in Video S1. Individual PSII subunits are color‐coded: PSII core in green, S trimers in cyan, LHCB5 in magenta, and LHCB4 in orange. Number of averaged projection maps for individual classes was as follows: 4681 (C_2_S_2_ “packed”), 2169 (C_2_S_2_ canonical), 2095 (C_2_S “packed”), 1935 (C_2_S canonical), 23,844 (CS), 2026 (C_2_)

The *lhcb4* mutant showed a markedly different pattern, lacking bands corresponding to large PSII assemblies (i.e., megacomplexes and C_2_S_2_M_2_ and C_2_S_2_M supercomplexes, Figure 3A). The largest PSII supercomplexes detected in the gel were assigned, according to their migration distance, to C_2_S_2_ and C_2_S supercomplexes. Due to their relatively low abundance, these supercomplex bands were clearly visible in the gel only in the chlorophyll fluorescence imaging mode, which is more sensitive than plain visible‐light image. Notably, fluorescence imaging further revealed that in *lhcb4*, unlike in WT, the band usually attributed to PSI/C_2_ is strongly fluorescent (Figure 3A). Closer inspection showed that, in agreement with previous studies on *lhcb4* mutants (Caferri et al. 2025; Guardini et al. 2022), this region forms a double band consisting of the PSI complex (weakly fluorescing at room temperature) and an adjacent co‐migrating band containing PSII complexes (highly fluorescing at room temperature) under the PSI complex. The PSII core complexes below PSI are either C_2_ dimers and/or CS monomers, both having migration distance similar to PSI.

To clarify how the absence of LHCB4 affects the structure of PSII (super)complexes, all three protein bands separated by CN‐PAGE from *lhcb4* mutant thylakoids were systematically analyzed by single‐particle EM. To our surprise, in the first, uppermost C_2_S_2_ band we identified two distinct supercomplex forms (Figure 3B). The less abundant form (see Figure S1) represents the canonical WT‐like C_2_S_2_ supercomplex with an empty LHCB4 binding site (Figures 3B,C). In this configuration, the S trimer lacks its usual interaction partner, which probably makes the complex highly fragile and difficult to isolate. This would explain its low abundance in CN‐PAGE (Figure 3A) and why it was not detected in previous studies of the *lhcb4* mutant. The more abundant form (see Figure S1) represents a LHCB4‐lacking C_2_S_2_ supercomplex with a more “packed” architecture (Figure 3B,C). A comparison with the newly observed WT‐like C_2_S_2_ lacking LHCB4 reveals that the “packed” C_2_S_2_ arises from a relative shift of the two CS monomers, with no major displacement of LHCB5 or the S trimers (Figure 3B,C; see also Video S1). This form probably corresponds to the PSII supercomplex structure previously described by de Bianchi et al. (2011). However, the lower resolution of their projection map likely prevented recognition of the shift in the C_2_ cores, leading the authors to attribute the structural difference to the altered positioning of LHCB5 and the S trimers in the absence of LHCB4. Since this “packed” C_2_S_2_ supercomplex was found to be the dominant form of our C_2_S_2_ band, it likely represents the more stable configuration of isolated C_2_S_2_ lacking LHCB4.

Single‐particle EM analysis of the second band from the *lhcb4* mutant, the C_2_S band, similarly revealed two C_2_ configurations (Figure 3B,C), with the “packed” form again more abundant (Figure S2).

Finally, single‐particle EM was performed also on the third band, corresponding to the highly fluorescent PSI/C_2_/CS double band described above (Figure 3A). Our analysis confirmed the presence of PSI particles in this region (Figure S3). However, the majority of the identified particles were CS monomers comprising a PSII core monomer, LHCB5, and one S LHCII trimer (Figure 3B,C). This CS form was much more prevalent than canonical C_2_ particles, with a CS to C_2_ ratio of ~17 in *lhcb4*, compared to ~0.8 in WT (Figure S4, see also Methods). Taken together, these data indicate that LHCB4 protein normally contributes to the stabilization of the interaction between PSII core monomers within the canonical C_2_S_2_ supercomplex. In its absence, PSII assemblies tend to reorganize either into the “packed” C_2_S_2_ configuration or, more frequently, to dissociate into CS supercomplexes.

### 3.3 Organization of PSII Supercomplexes in Thylakoid Membranes of the *lhcb4* Mutant

To determine whether the canonical and “packed” PSII core configurations identified in the *lhcb4* mutant using CN‐PAGE and single‐particle EM are also present in thylakoid membranes, we performed EM analysis of PSII cores directly in the membrane. PSII particles can be recognized in the membrane based on the high contrast provided by the oxygen‐evolving complex together with the lumenal domains of intrinsic core subunits. Single‐particle analysis of picked densities of PSII particles and successful fitting of the averaged PSII projections in the thylakoid membrane using the canonical WT‐like C_2_ model (Figures 4E and S5) indicates that only the canonical C_2_ configuration is present in *lhcb4* in situ. The “packed” C_2_S_(2)_ supercomplex was not detected in the membranes, which implies that it does not represent a stable physiological state, but reflects the structural instability of PSII supercomplexes lacking LHCB4, which manifests itself during isolation.

**FIGURE 4:**
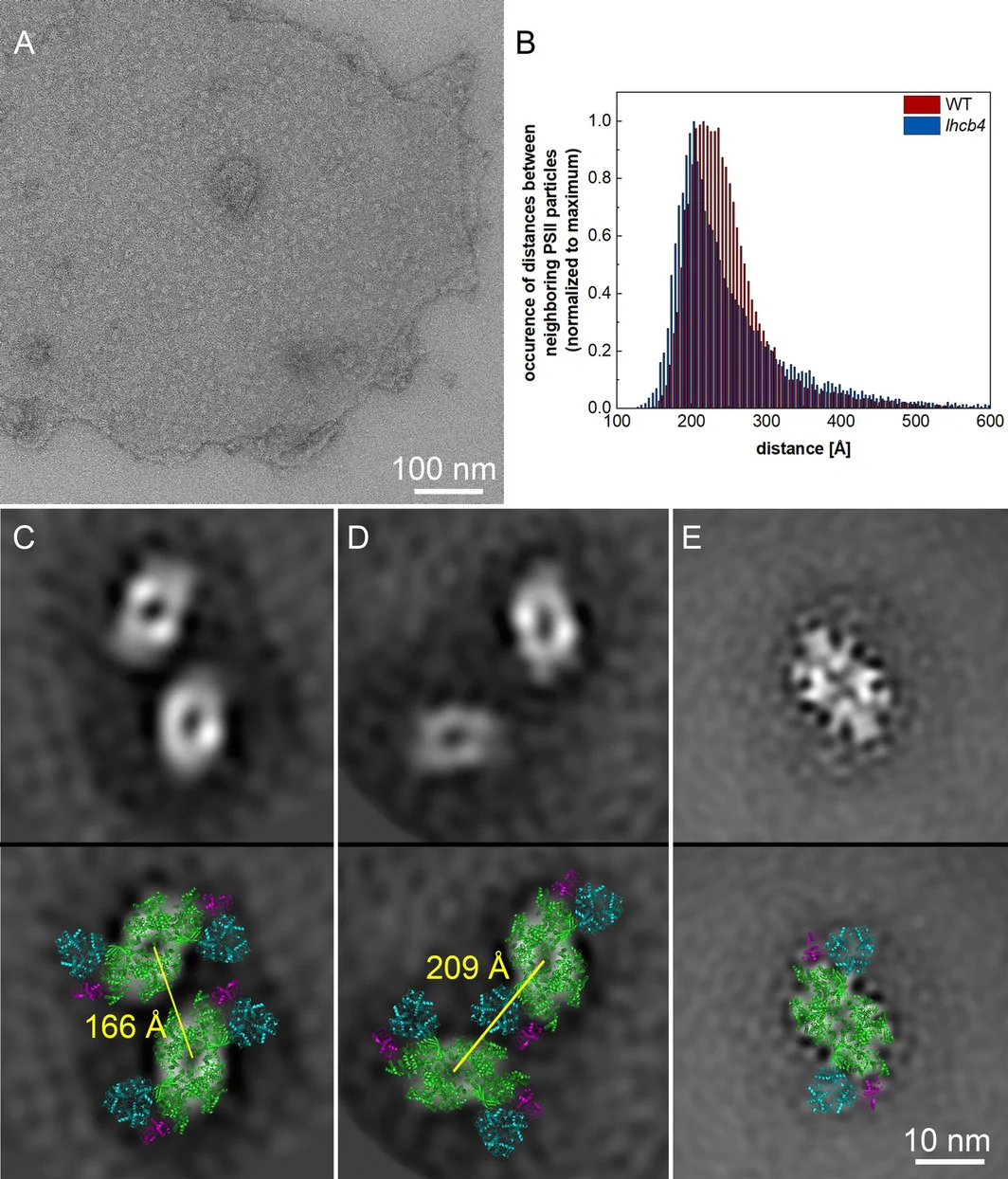
Organization of photosystem II in thylakoid membranes of the *lhcb4* mutant. (A) Example of electron micrographs of negatively stained thylakoid membrane isolated from the *lhcb4* mutant with densities corresponding to the PSII core complex. (B) Distribution of mutual distances between neighboring photosystem II particles in thylakoid membranes of WT and the *lhcb4* mutant. The distances between two closest neighboring PSII supercomplexes were analyzed using EM. Histograms are normalized to the maximum. Presented data for WT were adopted from Vánská et al. (2025). (C and D) Projection maps of PSII megacomplexes obtained using image analysis of PSII particles in thylakoid membranes. Two specific associations of PSII supercomplexes were found and fitted by the model of PSII supercomplex C_2_S_2_ without LHCB4 (see Figure 3C). Megacomplexes are averaged projections of 2325 (C) and 2247 (D). (E) Projection map of isolated PSII particle in the thylakoid membrane obtained as an average projection of 19,547 particles and fitted by the model of PSII supercomplex C_2_S_2_ without LHCB4 (see Figure 3B). Individual PSII subunits are color‐coded: PSII core in green, S trimers in cyan, and LHCB5 in magenta.

Using the coordinates of individually picked PSII particles, we analyzed the minimal distances between neighboring PSII particles within the membrane. This analysis revealed a narrower distribution of mutual distances between individual PSII particles in the membrane of *lhcb4* compared to WT. At the same time, most PSII particles in *lhcb4* thylakoids were closer to each other than in WT (peak distance at 202 Å in *lhcb4* and 217 Å in WT, Figure 4A,B). This result corresponds with the absence of M trimer in PSII supercomplexes in *lhcb4* mutant (Figure 3).

The observed closer spacing of PSII particles with WT‐like organization of PSII core dimers in *lhcb4* membranes prompted us to examine whether these particles form also higher‐order PSII assemblies (megacomplexes). In thylakoid membranes of plants, PSII supercomplexes are known to associate into megacomplexes through defined spatial arrangements of neighboring PSII supercomplexes in the membrane plane (Kouřil et al. 2020; Nosek et al. 2017; Shan et al. 2024). We therefore analyzed the local membrane environment around individually picked PSII particles and searched for specifically associated neighboring PSII particles within the *lhcb4* membrane. This analysis revealed two distinct types of PSII megacomplexes (Figures 4C,D), characterized by inter‐PSII distances of 166 and 209 Å. However, a majority of PSII particles in the *lhcb4* membrane do not participate in the megacomplex formation, as no specific spatial association with neighboring complexes was detected for most particles (Figure 4E).

Both revealed PSII megacomplexes in the *lhcb4* are formed by nonparallel associations of PSII supercomplexes (Figures 4C,D). Fitting of the projection maps of the megacomplexes demonstrates that the observed assemblies are compatible with two C_2_S_2_ supercomplexes lacking LHCB4 (Figure 4C,D). The fitting with larger supercomplexes, such as C_2_S_2_M_2_, would lead to steric clashes of the M trimer, indicating that the PSII megacomplexes are formed exclusively by C_2_S_2_ and mediated primarily by LHCB5. The identified nonparallel associations are consistent with our previous study on WT Arabidopsis (Nosek et al. 2017), which described interactions between PSII supercomplexes mediated by LHCB5, and with the absence of such nonparallel associations of PSII supercomplexes in the *lhcb5* mutant (Vánská et al. 2025). As no PSII megacomplexes were detected in CN‐PAGE of the *lhcb4* mutant (Figure 3A), we conclude that the megacomplexes lacking LHCB4 observed in thylakoid membranes are too labile to be preserved as distinct bands in CN‐PAGE.

### 3.4 Low Stability of PSII Core Dimer Lacking All Minor Antenna Proteins

The higher abundance of CS supercomplexes compared to C_2_S_2_ obtained using CN‐PAGE in the *lhcb4* mutant (Figure 3) suggests that the interaction between monomeric cores in the C_2_ dimer lacking LHCB4 might be weaker than the attachment of the S trimer to the monomeric core via LHCB5. The formation of the “packed” C_2_S_2_ with shifted CS monomers (Figure 3 and Video S1) supports this view. In order to estimate the structural stability of the “packed” C_2_S_2_, we fitted the electron density map of this complex with a model composed of two shifted CS monomers and searched for potential steric clashes between the CS monomers. In this rigid‐body fitting, the S‐LHCII trimer of one CS unit approaches the PSII core subunits of the neighboring CS monomer very closely, leading to clashes (Figure S6). Because the “packed” complex is clearly present in the EM data, there must be small local displacements of interacting subunits in this structure that are not captured in the rigid structural model. Such adjustments are nevertheless likely to introduce structural strain or suboptimal packing within the dimeric assembly. This reduced structural compatibility may facilitate dissociation of the “packed” C_2_S_2_ into monomeric CS complexes during isolation, as observed in the CN‐PAGE analysis of the *lhcb4* mutant (Figure 3). This scenario could mean that the presence of LHCB5, forming highly stable CS monomers, could be responsible for the disruption of the PSII core dimer in the absence of LHCB4.

A question arises as to whether the PSII core dimer will also be unstable in the absence of both LHCB5 and LHCB4 antenna proteins. To address this possibility, we have analyzed the stability of PSII core dimer in the NoM Arabidopsis mutant, which lacks LHCB4, LHCB5, and implicitly also LHCB6 (Dall'Osto et al. 2014). As expected, CN‐PAGE of the NoM samples showed no PSII supercomplexes above the PSI/C_2_/CS band (Figure 5A), and single‐particle EM analysis of the PSI/C_2_/CS band confirmed that PSII is present exclusively in the C_2_ form without CS particles (Figure S7). This observation agrees with the inability of C_2_ to bind the S trimer in the absence of monomeric antenna proteins.

**FIGURE 5:**
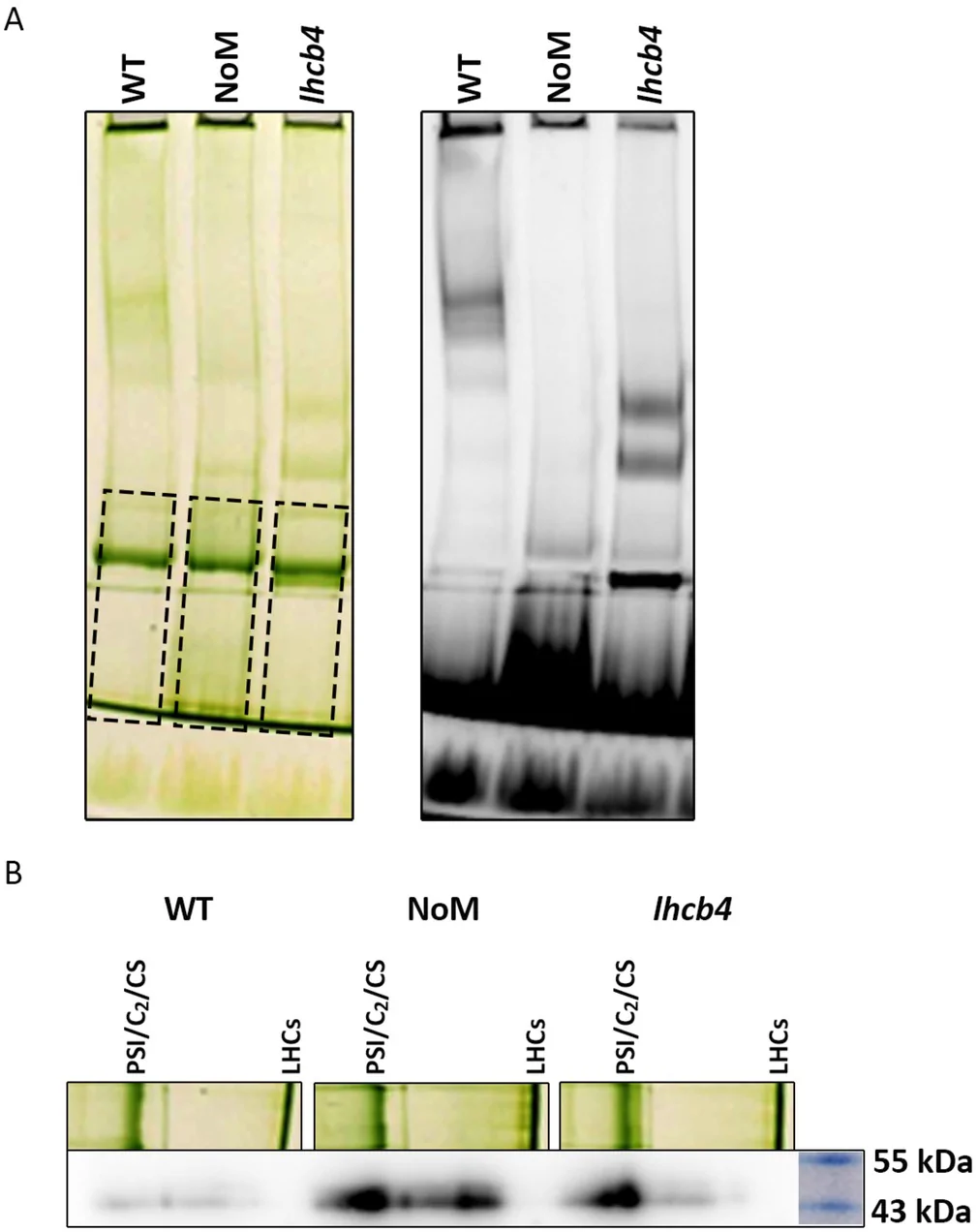
Separation and analysis of pigment–protein complexes from *Arabidopsis thaliana* WT and its two mutants NoM and *lhcb4*. (A) CN‐PAGE separation of pigment‐protein complexes from the thylakoid membranes of WT, NoM, and *lhcb4*. The image shows the selected gel lines scanned at room temperature in transmission mode (color image) and in fluorescence mode (black and white image). (B) Immunoblot identification of the PSBB (CP47) subunit on a PVDF membrane with transferred proteins from the second‐dimension denaturing PAGE. Two horizontal bands of the marker (blue color) designate the molecular weight of proteins at these positions.

To determine whether C_2_ is stable or whether it is prone to disintegration into core monomers in NoM, we performed 2D‐PAGE analysis of the lower CN‐PAGE regions and compared them with WT and the *lhcb4* mutant (Figure 5). The disintegration of C_2_, monitored by immunoblotting against the CP47 subunit, was pronounced in NoM compared to *lhcb4* and WT (Figure 5B). In WT, a very weak CP47 signal in and below the PSI/C_2_/CS band clearly shows that the majority of PSII is present in larger supercomplexes (Figure 3A). In *lhcb4*, the PSI/C_2_/CS band contains a large amount of CP47 because of the abundant CS complexes (Figures 3A and S3), but the absence of a strong signal below this band shows that C_2_/CS is not largely disintegrated. In contrast, NoM shows a high amount of CP47 in the PSI/C_2_/CS band as well as below this band (Figure 5B), indicating pronounced disintegration of C_2_ into smaller fragments. Taken together, these results lead to the conclusion that LHCB4 itself plays a crucial structural role in maintaining the stability of the PSII core dimer, likely through its stromal‐loop‐mediated interactions with PSII core subunits.

### 3.5 Stability and Function of PSII Assemblies in the *lhcb4* Mutant In Vivo

Our analysis revealed that PSII supercomplexes lacking LHCB4 exhibit reduced stability and tend to rearrange/dissociate even under relatively mild disturbance, e.g., during solubilization of thylakoid membranes for CN‐PAGE (Figure 3). Our next question was whether this instability occurs in vivo, and to test this, we applied gradual heating to intact leaves and assessed PSII assembly stability using circular dichroism (CD) spectroscopy (Garab and van Amerongen 2009).

In general, the dominant CD bands (ψ‐type) observed in leaves, chloroplasts, and isolated grana membranes arise from long‐range dipole–dipole interactions between chlorophylls and carotenoids within PSII and LHCII complexes (Garab 2014, 2016; Garab and van Amerongen 2009; Lambrev and Akhtar 2019). It is well established that the amplitudes of the positive bands peaking at approximately 505 and 685 nm reflect both LHCII content and long‐range lateral macro‐organization of PSII supercomplexes (Tóth et al. 2016). We observed a pronounced reduction in both ψ‐type CD band amplitudes in *lhcb4* relative to WT (Figure 6A). Since the abundance of LHCII in *lhcb4* is comparable to that in WT (Figure 1D), this decrease can be interpreted by the reduced extent of lateral macro‐organization of PSII supercomplexes in thylakoid membranes due to LHCB4 absence. Consistently, *lhcb4* exhibited lower thermal stability of these CD bands (Figures 6B–D and Table S2), revealing markedly reduced heat tolerance of PSII macro‐organization.

**FIGURE 6:**
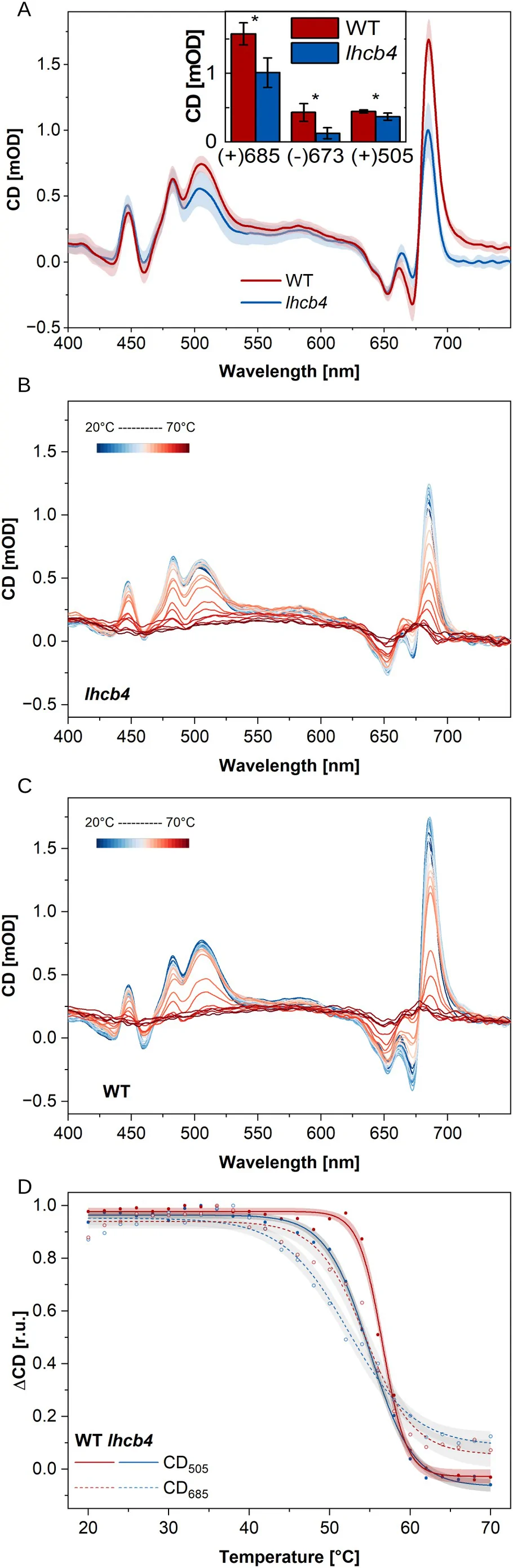
Circular dichroism (CD) spectra of leaves from wild type (WT) and the *lhcb4* mutant. (A) The CD spectra of leaves from wild type (WT) (red) and the *lhcb4* mutant (blue) normalized to the Q_y_ absorbance maximum. Average spectra from five leaves are represented as a solid line and the shadows represent the standard deviation. The inset presents the amplitudes of ψ‐type CD bands at wavelengths of (+)685 nm, (−)673 nm and (+)505 nm, means ± SD (*n* = 5). Asterisks indicate statistically significant differences between *lhcb4* and WT (*p* = 0.05). Temperature dependence of the CD spectra of intact leaves from dark adapted *lhcb4* mutant (B) and WT (C) plants, measured in the temperature range 20°C–70°C in 2°C increments. Temperature dependences of the intensity of the two positive ψ‐type CD bands (D) for *lhcb4* mutant and WT plants. The data points in (D) are normalized to the maximal values. Data points are fitted with a sigmoidal curve (solid lines); 95% confidence intervals are represented by shadows. Mean values and SD of transition temperatures (Tm) from five independent experiments are presented in Table S2.

The decreased thermal stability of PSII assemblies in *lhcb4* as deduced from CD spectroscopy nicely corresponds with impaired heat tolerance of PSII photochemistry, as evidenced by changes in chlorophyll FTCs (Figure S8). During gradual heating of leaf segments, *lhcb4* exhibited an earlier and steeper increase in the fluorescence F_O_ level than the WT. This shows that the heat‐induced accumulation of Q_A_^−^ resulting from PSII conformational changes/thermal damage occurs in *lhcb4* at lower temperatures than in WT (e.g., Schreiber and Armond 1978; Kouřil et al. 2004; Lípová et al. 2010, and references therein).

In summary, our results demonstrate that LHCB4 plays an essential role not only in mediating LHCII attachment to PSII, but also in maintaining the intrinsic stability of the PSII core dimer. In the absence of LHCB4, PSII dimer assemblies show increased vulnerability and readily destabilize during isolation, resulting in the formation of aberrant “packed” supercomplexes and in monomerization, although wild‐type‐like organization of the PSII dimer is largely preserved in thylakoid membranes. Despite this preserved architecture in situ, the absence of LHCB4 affects PSII function, as evidenced by affected charge recombination pathways within the PSII core. This structural vulnerability of PSII assemblies is manifested in vivo as lower heat stability of lateral PSII macro‐organization and PSII photochemistry.

## Author Contributions

T.V., P.I., and R.K. planned and designed the research, all authors performed experiments and analyzed the data. T.V., I.I., R.K., and P.I. wrote the manuscript with input from all authors, and all authors revised and approved it.

## Funding

This work was supported by the Czech Science Foundation (21‐05497S), the Palacký University (IGA_PrF_2026_022), the ERDF Programme Johannes Amos Comenius—TowArds Next GENeration Crops (CZ .02.01.01/00/22_008/0004581), and the Operational Programme Just Transition—LERCO project (CZ.10.03.01/00/22_003/0000003). We acknowledge CEITEC Proteomics Core Facility of CIISB, Instruct‐CZ Centre, supported by MEYS CR (LM2023042) and e‐INFRA CZ (ID:90254).

## Conflicts of Interest

The authors declare no conflicts of interest.

## Supporting information


**Table S1:** Physiological parameters of Arabidopsis WT and *lhcb4* mutant plants.
**Table S2:** Transition temperatures (Tm) ‐ CD bands for wild‐type (WT) and *lhcb4* mutant.
**Figure S1:** Single‐particle analysis and 2D classification of C_2_S_2_ band from the *lhcb4* mutant (Figure 3A). Numbers of averaged projections in given classes are indicated.
**Figure S2:** Single‐particle analysis and 2D classification of C_2_S band from the *lhcb4* mutant (Figure 3A). The image represents the selection of the most abundant classes. Numbers of averaged projections in given classes are indicated.
**Figure S3:** Single‐particle analysis and 2D classification of PSI/C_2_/CS band from the *lhcb4* mutant (Figure 3A). The image represents the selection of the most abundant classes. Numbers of averaged projections in given classes are indicated. Classes with numbers 4336, 4274, 2101, and 1853 are variously tilted projection maps of PSI complexes.
**Figure S4:** Single‐particle analysis and 2D classification of PSI/C_2_/CS band from the WT Arabidopsis (Figure 3A). The image represents the selection of the most abundant classes. Numbers of averaged projections in given classes are indicated. Classes with numbers 4931, 4587, 4542, 3112, 2801, 2589, 2411, 1955, 1887, 1748, 1591, 1547, 1370, 1239, 1171, 1144, 1104, 967, 755, 689, 628, and 381 are variously tilted projection maps of PSI complexes.
**Figure S5:** Comparison of canonical and “packed” PSII core dimer models with the averaged projection map of PSII in the thylakoid membrane of the *lhcb4* mutant. The average projection map of PSII particles in thylakoid membranes of the *lhcb4* mutant (the same projection map as in Figure 4E) was overlaid with structural models of the PSII core dimer (C_2_) representing (A) the canonical C_2_ arrangement and (B) the “packed” C_2_ arrangement (for the models see Figure 3C). The canonical model closely corresponds to the density map of the C_2_, whereas the “packed” model shows a clear discrepancy, confirming the presence of the canonical C_2_ organization in the thylakoid membrane of the *lhcb4* mutant.
**Figure S6:** Steric clashes in the “packed” C_2_S_2_ supercomplex from the *lhcb4* mutant. Structural rearrangement of C_2_S_2_ supercomplex from the canonical to the “packed” configuration in the *lhcb4* mutant leads to steric clashes between the S‐LHCII trimers and PSII core subunits (PSBB and PSBH). These clashes, highlighted in magenta, were detected as interactions with interatomic distances ≤ 2 Å. The clashes appearing between the S‐LHCII antenna and core components in the packed configuration likely destabilize the C_2_S_2_ supercomplex. Steric clashes were evaluated in PyMOL Molecular Graphics System (Version 3.1.4.1, Schrödinger LLC.) using an interatomic distance cutoff of 2 Å.
**Figure S7:** Single‐particle analysis and 2D classification of PSI/C_2_/CS band from the NoM mutant (see representative gel figure in Figure 5A). The image represents the selection of the most abundant classes. Numbers of averaged projections in given classes are indicated.
**Figure S8:** Chlorophyll fluorescence temperature curves in Arabidopsis WT and *lhcb4* mutant. Detached leaves were heated from room temperature to 70°C in a water bath at a rate of 2°C min^−1^. Simultaneously, chlorophyll fluorescence intensity under weak excitation (F_O_ level, minimal fluorescence) was recorded (see Ilík et al. 2018). Leaves were dark‐adapted for 30 min. The maximum curvature points (*T*_C_) of the initial fluorescence increase are indicated. Insert: The mean of T_C_ and SD for WT and *lhcb4*. Representative curves are presented. Number of repetitions was 5–6.

**Video S1:** Comparison of electron density maps of “canonical” and “packed” C_2_S_2_ supercomplex particles separated by CN‐PAGE from thylakoid membranes of the *lhcb4* mutant. Individual C_2_S_2_ supercomplex electron density maps were taken from Figure 3B. This presentation supports the view that the “packed” C_2_S_2_ particle is formed by a shift of CS parts in the canonical C_2_S_2_ supercomplex lacking LHCB4.

## Data Availability

Datasets were deposited into a publicly accessible repository via Zenodo under 10.5281/zenodo.19388327. Mass spectrometry proteomics data were deposited to the ProteomeXchange Consortium via PRIDE partner repository under dataset identifier PXD076442.
