# Supplementary material for: Light‐Harvesting Protein LHCB4 Is Important for Stable Dimeric Organization of Photosystem II
Source: Physiol Plant. 2026 Oct 9;178(5):e71144. doi: 10.1111/ppl.71144 (PMC13651784; doi:10.1111/ppl.71144)
Supplement: Supplementary file 1 [file PPL-178-e71144-s002.pdf]

SUPPORTING INFORMATION

Manuscript title: Light-harvesting protein LHCB4 is important for stable dimeric organization of photosystem II

Authors: Tereza Vánská, Monika Opatíková, Roman Kouřil, Zuzana Kučerová, Václav Karlický, Iva Ilíková, Marek Rác, Pavel Roudnický, Vojtěch Chýlek, Petr Ilík

Content of supporting material:

Method S1

Table S1, S2

Figure S1, S2, S3, S4, S5, S6, S7

Video S1

Method S1 Sample preparation for mass spectrometry analysis and data processing

Proteins from *Arabidopsis thaliana* samples (thylakoid membranes) were extracted in SDT buffer (4% SDS, 0.1M DTT, 0.1M Tris/HCl, pH 7.6) in a thermomixer (Eppendorf ThermoMixer C, 30min, 95°C, 750 rpm). After that, all samples were centrifuged (15 min, 20 000 x g) and the supernatants were each divided to 3 parts (each ca 75 µg of total protein) and used for filter-aided sample preparation (FASP) as described elsewhere [1] using 0.75 µg of trypsin (sequencing grade; Sigma-Aldrich). The resulting peptides were extracted into LC-MS vials by 2.5% formic acid (FA) in 50% acetonitrile (ACN) and 100% ACN with the addition of polyethylene glycol (final concentration 0.001%) [2] and concentrated in a SpeedVac concentrator (Thermo Fisher Scientific).

LC-MS/MS analyses of all peptides were done using UltiMate 3000 RSLCnano system (Thermo Fisher Scientific) connected to Orbitrap Exploris 480 spectrometer (Thermo Fisher Scientific). Prior to LC separation, tryptic digests were online concentrated and desalted using trapping column (Acclaim PepMap 100 C18, dimensions 300 µm ID, 5 mm long, 5 µm particles, Thermo Fisher Scientific). After washing of trapping column with 0.1% FA, the peptides were eluted in backflush mode (flow 400 nl min^{-1}) from the trapping column onto an analytical column separation column (EASY-Spray column, 75µm ID, 250 mm long, 2 µm particles, Thermo Fisher Scientific) by 90 min gradient program (flow rate 250 nl min^{-1}, 5-37% of mobile phase B; mobile phase A: 0.1% FA in water; mobile phase B: 0.1% FA in 80% ACN). Both columns were heated to 40°C.

MS data were acquired in a data-dependent strategy (cycle time 2.5s). Survey scan range was set to m/z 350-2000 with the resolution of 120 000 (at m/z 200), normalized target value of 250 % and maximum injection time of 500 ms. HCD MS/MS spectra (isolation window m/z 1.2, 30% relative fragmentation energy) were acquired from 120 m/z with a relative target value of 50 % (intensity threshold 5×103), resolution of 15 000 (at m/z 200) and maximum injection time of 50 ms. Dynamic exclusion was enabled for 45 s.

For data evaluation, we used MaxQuant software (v2.0.3.0) [3] with inbuild Andromeda search engine [4]. Search was done against protein databases of Arabidopsis thaliana (27 474 protein sequences, version from 2022-08-07, based on ftp://ftp.uniprot.org/pub/databases/uniprot/current_release/knowledgebase/reference_proteomes/Eukaryota/UP000006548/UP000006548_3702.fasta.gz) and cRAP contaminants (112 sequences, version from 2018-11-22, based on http://www.thegpm.org/crap). Modifications were set as follows for database search: oxidation (M), deamidation (N, Q), and acetylation (Protein N-term) as variable modifications, with carbamidomethylation (C) as a fixed modification. Enzyme specificity was tryptic with two permissible miscleavages. Only peptides and proteins with false discovery rate threshold under 0.01 were considered. Relative protein abundance was assessed using protein intensities calculated by MaxQuant. Intensities of reported proteins were further evaluated using software container environment (https://github.com/OmicsWorkflows/KNIME_docker_vnc; version 4.6.3a). Processing workflow is available upon request and it covers, in short, reverse hits and contaminant protein groups (cRAP) removal, protein group intensities log2 transformation and normalization (loessF). For the purpose of this article, protein groups reported by MaxQuant are referred to as proteins.

Mass spectrometry proteomics data were deposited to the ProteomeXchange Consortium via PRIDE [5] partner repository under dataset identifier PXD076442.

References:

1) Wiśniewski JR, Zougman A, Nagaraj N, Mann M. Universal sample preparation method for proteome analysis. Nat Methods. 2009; 6: 359–362. https://doi.org/10.1038/nmeth.1322

2) Stejskal, K., Potěšil, D. & Zdráhal, Z. Suppression of Peptide sample losses in autosampler vials. J. Proteome Res. 12, 3057–3062 (2013).

3) Cox J, Mann M. MaxQuant enables high peptide identification rates, individualized p.p.b.-range mass accuracies and proteome-wide protein quantification. Nat Biotechnol. 2008; 26: 1367–1372. https://doi.org/10.1038/nbt.1511

4) Cox J, Neuhauser N, Michalski A, Scheltema RA, Olsen J V., Mann M. Andromeda: A Peptide Search Engine Integrated into the MaxQuant Environment. J Proteome Res. 2011; 10: 1794–1805. https://doi.org/10.1021/pr101065j
5) Perez-Riverol Y, Csordas A, Bai J, Bernal-Llinares M, Hewapathirana S, Kundu DJ, et al. The PRIDE database and related tools and resources in 2019: improving support for quantification data. Nucleic Acids Res. 2019; 47: D442–D450. https://doi.org/10.1093/nar/gky1106

Supporting Tables

Table S1. Physiological parameters of Arabidopsis WT and *lhcb4* mutant plants.

	WT	*lhcb4*
fresh weight of the rosette [mg]	1201 ± 130 [a]	1165 ± 83 [a]
chlorophyll a + b [µg cm^{-2}]	18.7 ± 1.1 [a]	18.5 ± 0.9 [a]
chlorophyll a/b ratio	2.95 ± 0.02 [a]	2.98 ± 0.10 [a]
carotenoids [µg cm^{-2}]	3.09 ± 0.21 [a]	3.11 ± 0.18 [a]
A [µmol CO_2 m^{-2} s^{-1}]	2.97 ± 0.27 [a]	2.62 ± 0.43 [a]
F_O	421 ± 21 [a]	496 ± 21 [b]
F_M	2187 ± 117 [a]	2056 ± 109 [a]
F_V/F_M	0.807 ± 0.004 [a]	0.759 ± 0.005 [b]
TR_O/RC	1.548 ± 0.067 [a]	1.522 ± 0.019 [a]

Notes: Presented values are means ± SD. Fresh weight of individual rosettes was measured for n = 7. Pigment content is expressed in µg per cm^2 of the leaf area (n = 6). Net assimilation rate (A) was measured for n = 6. Chlorophyll fluorescence parameters (F_O, F_M, F_V/F_M and TR_0/RC) were measured for n = 5. Data within a row followed by the same letter are not significantly different (P > 0.05).

Table S2 Transition temperatures (Tm) of selected CD bands for wild-type (WT) and lhcb4 mutant.

CD signal [nm]	WT	*lhcb4*
685–730	54.4 ± 0.4°C [a]	52.3 ± 0.9 °C [b]
505–550	56.6 ± 0.8°C [a]	54.9 ± 0.7 °C [b]

Notes: The intact leaves were gradually heated in the range between 20 °C and 70 °C. The amplitudes for the individual bands were calculated from the difference in the intensity at specific wavelengths. Tm is defined as the temperature at which the intensity of the CD band is decreased to 50% of its value at 20 °C. Mean values and standard deviations from five independent experiments are presented. Data within a row followed by the same letter are not significantly different (P > 0.05).

Supporting Figures

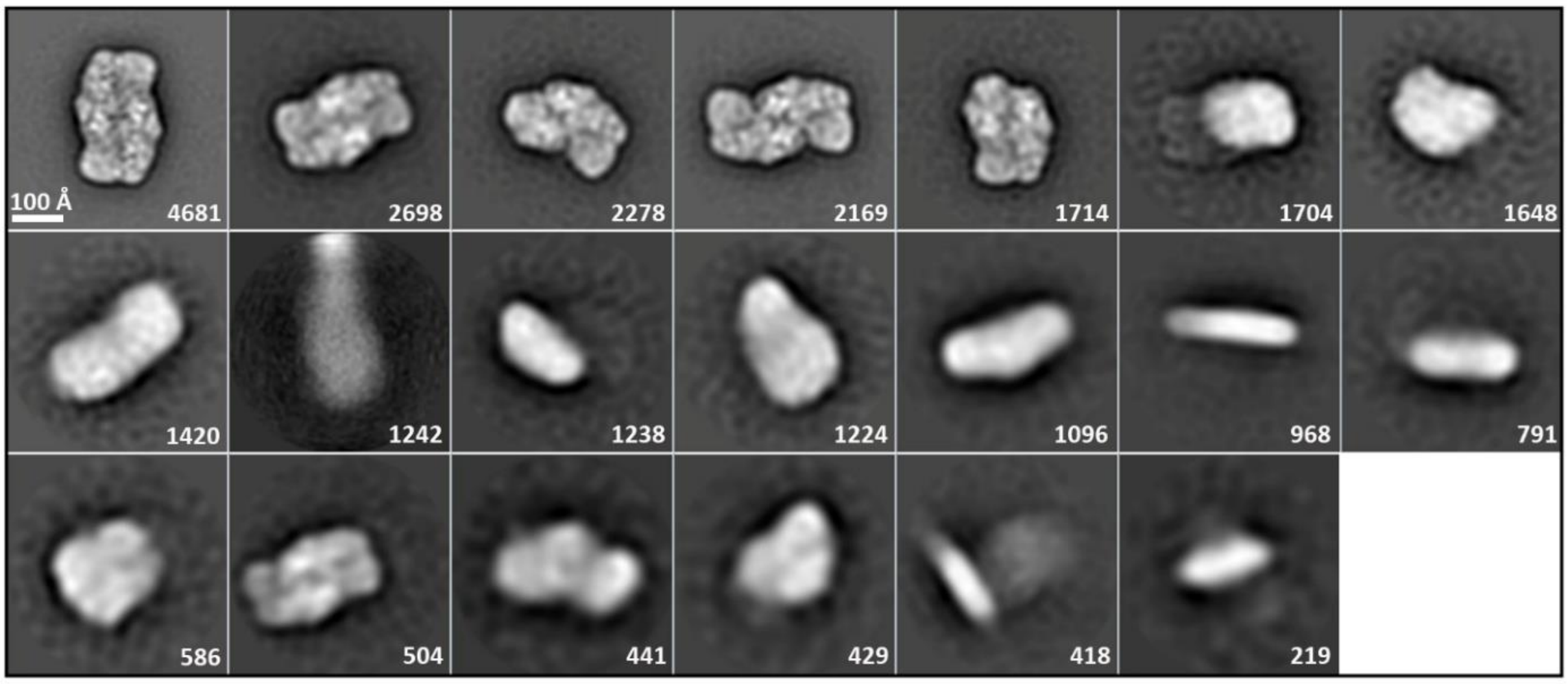

Figure S1 Single-particle analysis and 2D classification of C_2S_2 band from the *lhcb4* mutant (Figure 3A). Numbers of averaged projections in given classes are indicated.

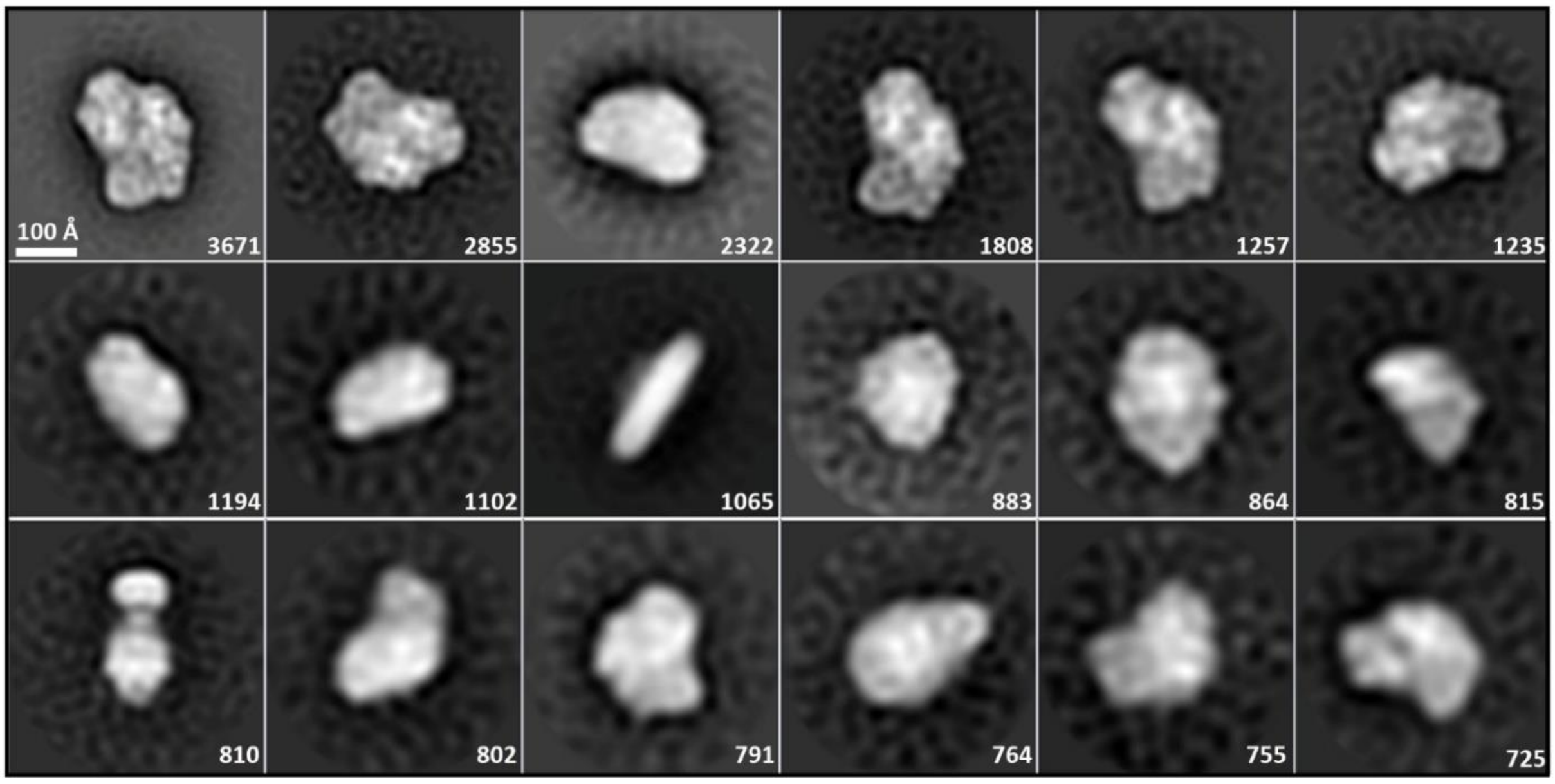

Figure S2 Single-particle analysis and 2D classification of C_2S band from the *lhcb4* mutant (Figure 3A). The image represents the selection of the most abundant classes. Numbers of averaged projections in given classes are indicated.

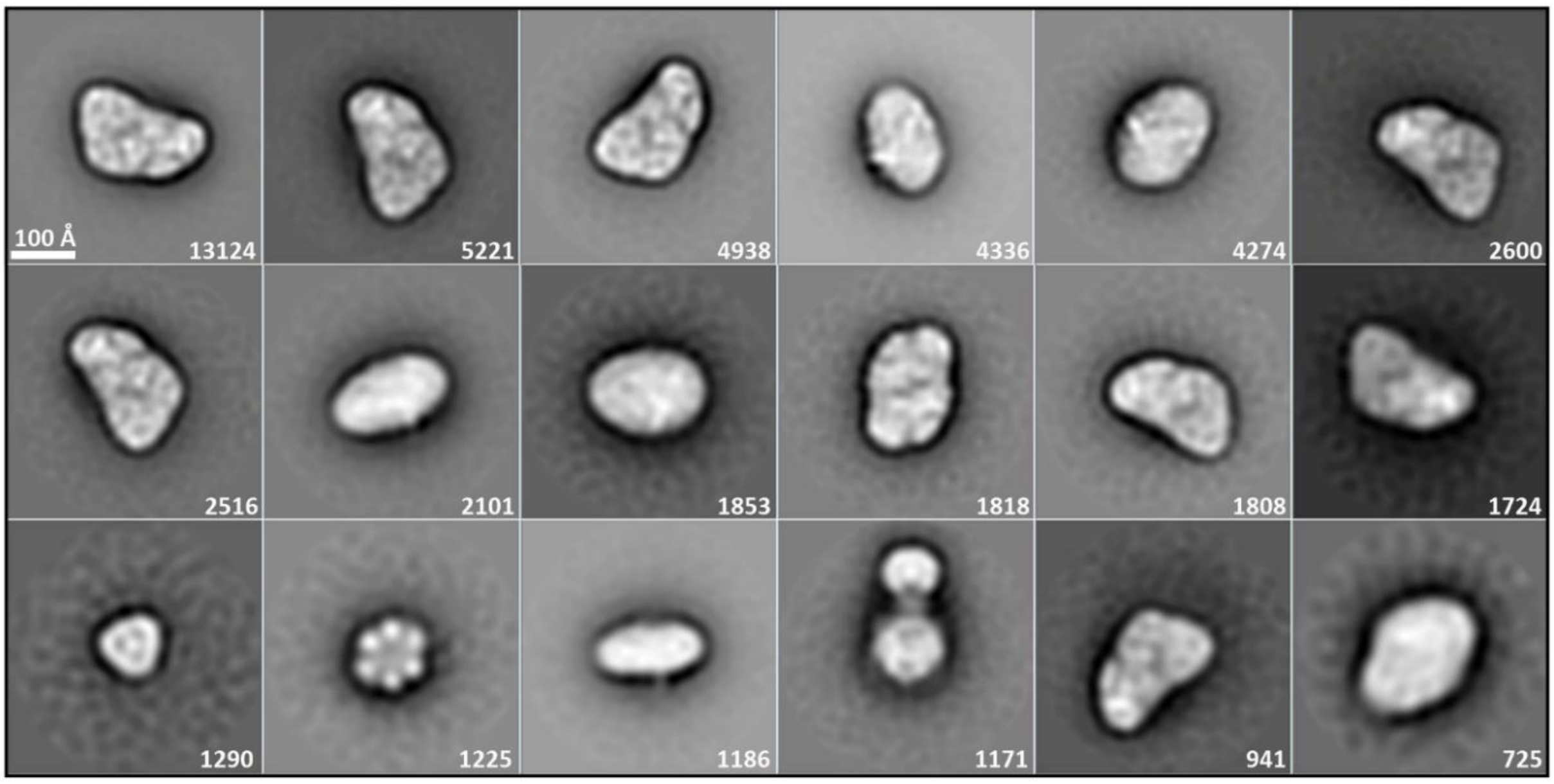

Figure S3 Single-particle analysis and 2D classification of PSI/C_2/CS band from the *lhcb4* mutant (Figure 3A). The image represents the selection of the most abundant classes. Numbers of averaged projections in given classes are indicated. Classes with numbers 4336, 4274, 2101 and 1853 are variously tilted projection maps of PSI complexes.

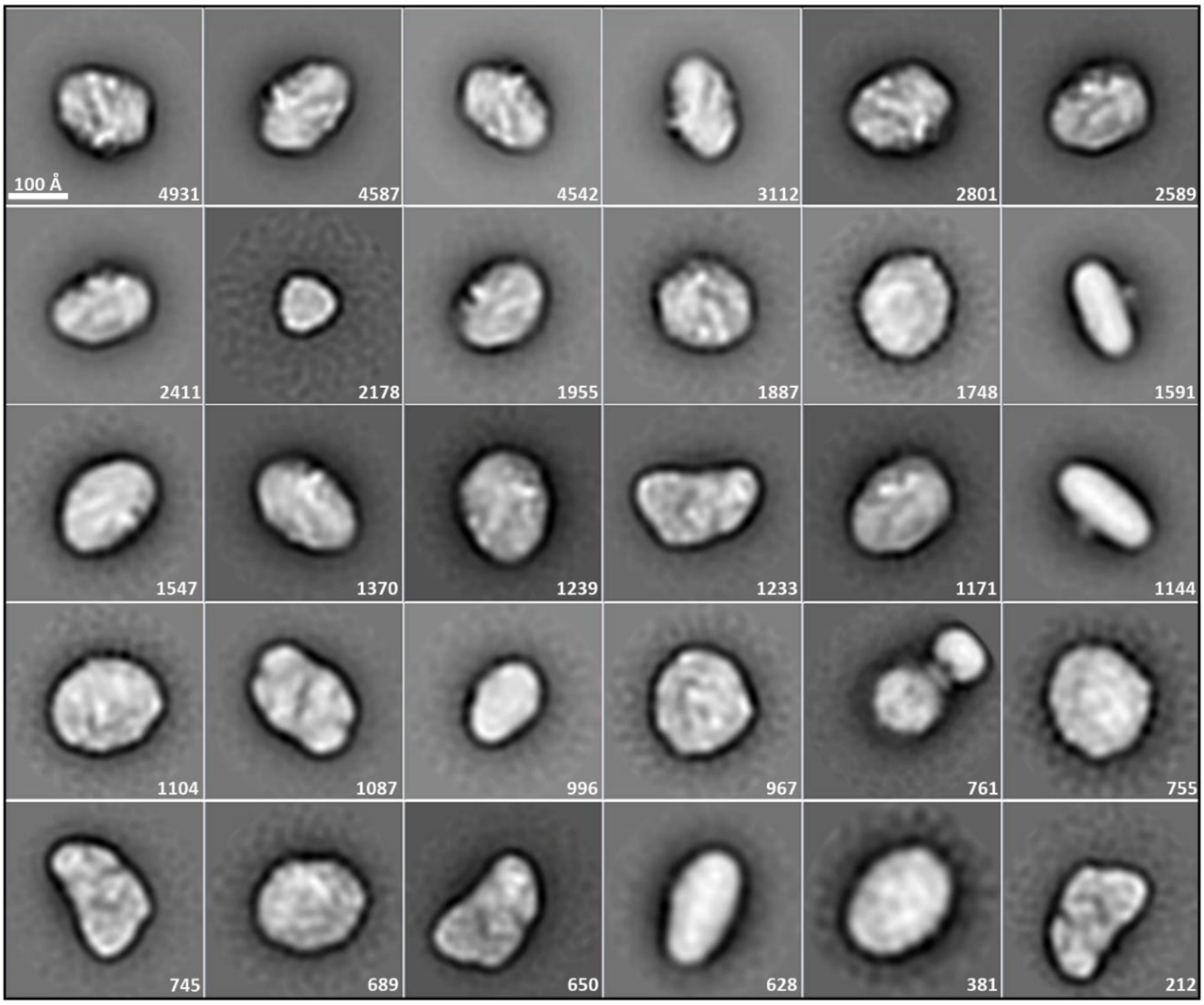

Figure S4 Single-particle analysis and 2D classification of PSI/C_2/CS band from the WT Arabidopsis (Figure 3A). The image represents the selection of the most abundant classes. Numbers of averaged projections in given classes are indicated. Classes with numbers 4931, 4587, 4542, 3112, 2801, 2589, 2411, 1955, 1887, 1748, 1591, 1547, 1370, 1239, 1171, 1144, 1104, 967, 755, 689, 628 and 381 are variously tilted projection maps of PSI complexes.

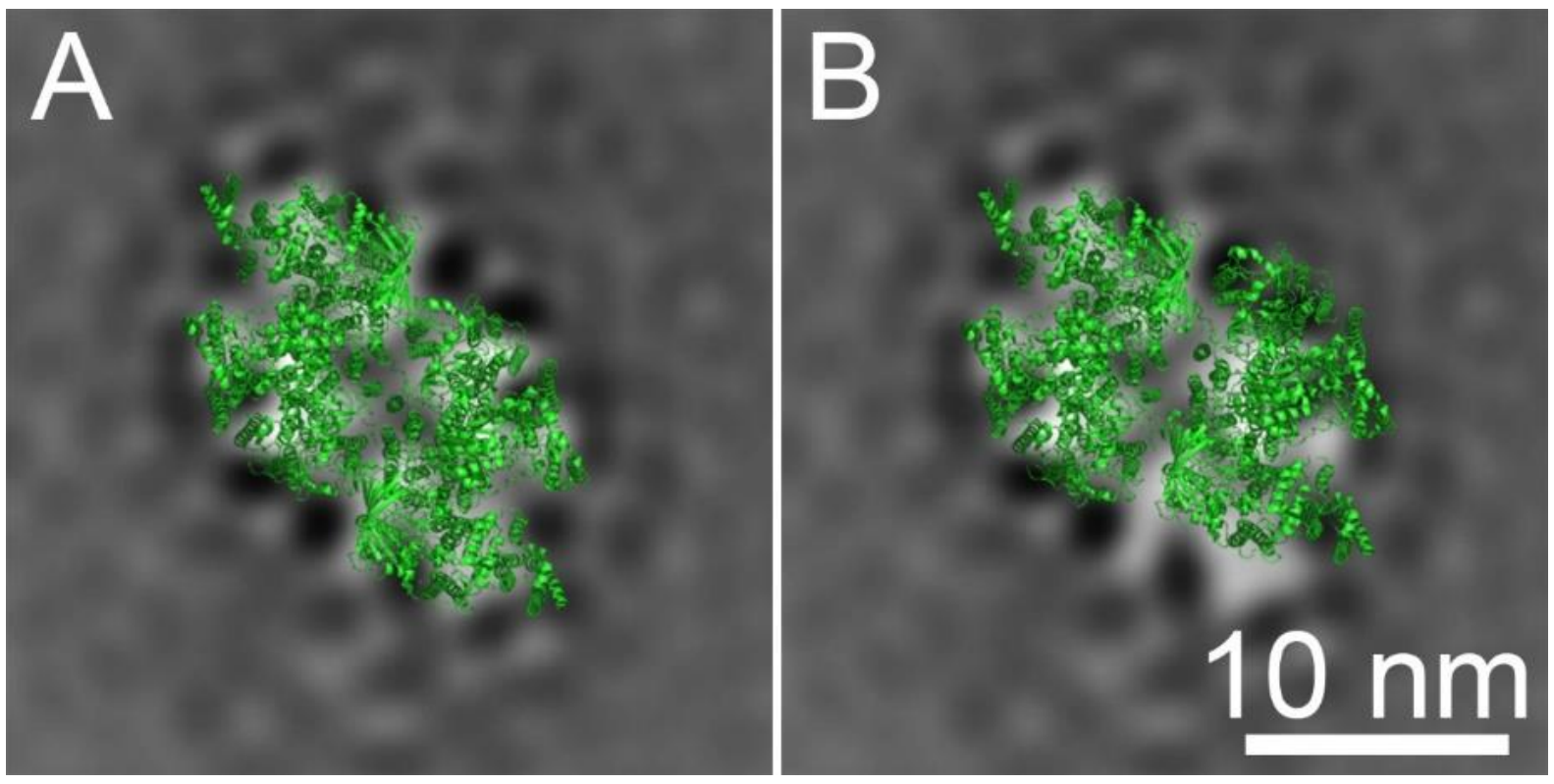

Figure S5 Comparison of canonical and 'packed' PSII core dimer models with the averaged projection map of PSII in the thylakoid membrane of the *lhcb4* mutant. The average projection map of PSII particles in thylakoid membranes of the *lhcb4* mutant (the same projection map as in Figure 4E) was overlaid with structural models of the PSII core dimer (C_2) representing (A) the canonical C_2 arrangement and (B) the 'packed' C_2 arrangement (for the models see Figure 3C). The canonical model closely corresponds to the density map of the C_2, whereas the 'packed' model shows a clear discrepancy, confirming the presence of the canonical C_2 organization in the thylakoid membrane of the *lhcb4* mutant.

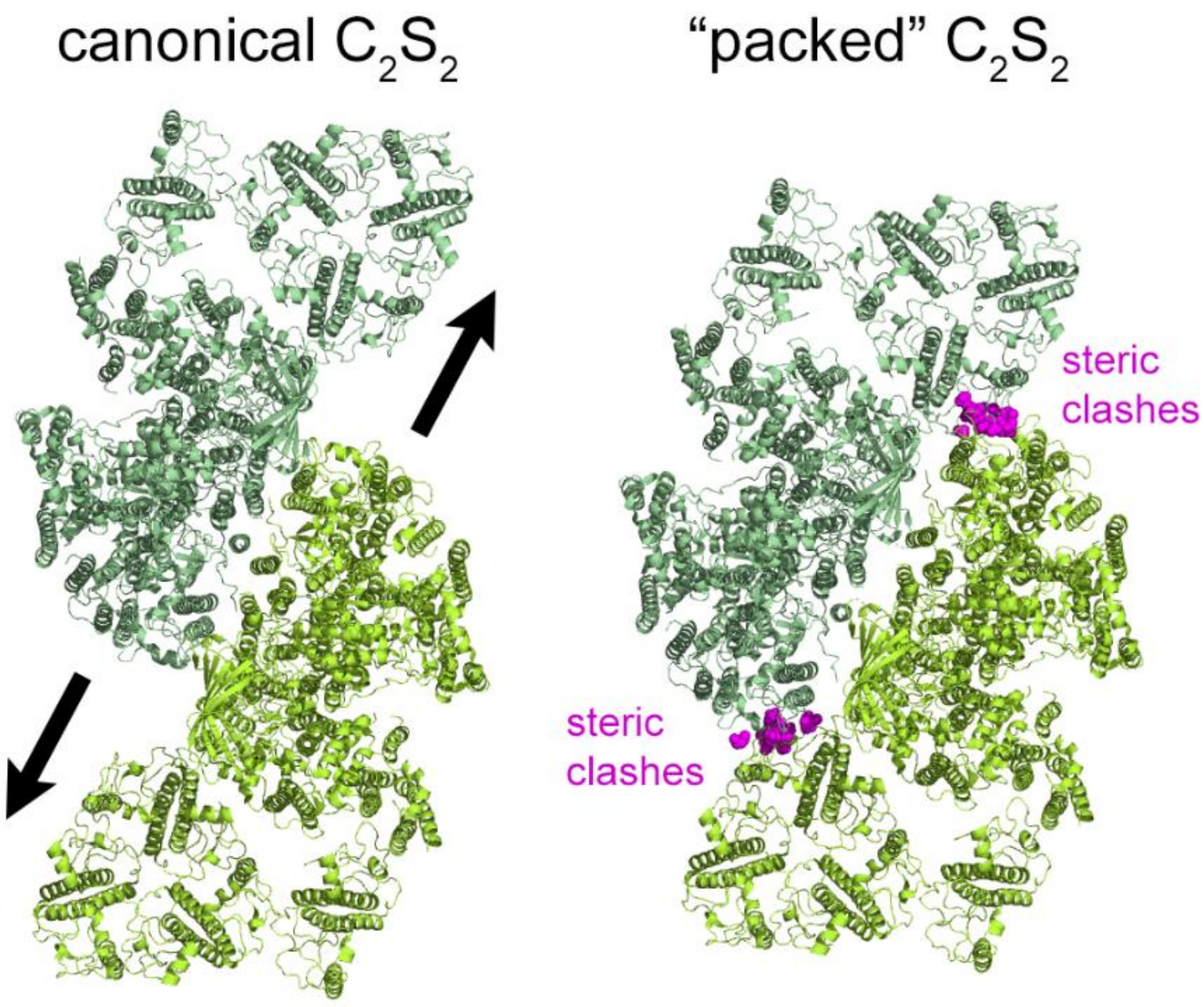

Figure S6 Steric clashes in the "packed" C_2S_2 supercomplex from the *lhcb4* mutant. Structural rearrangement of C_2S_2 supercomplex from the canonical to the "packed" configuration in the *lhcb4* mutant leads to steric clashes between the S-LHCII trimers and PSII core subunits (PSBB and PSBH). These clashes, highlighted in magenta, were detected as interactions with interatomic distances ≤ 2 Å. The clashes appearing between the S-LHCII antenna and core components in the packed configuration likely destabilize the C_2S_2 supercomplex. Steric clashes were evaluated in PyMOL Molecular Graphics System (Version 3.1.4.1., Schrödinger, LLC.) using an interatomic distance cutoff of 2 Å.

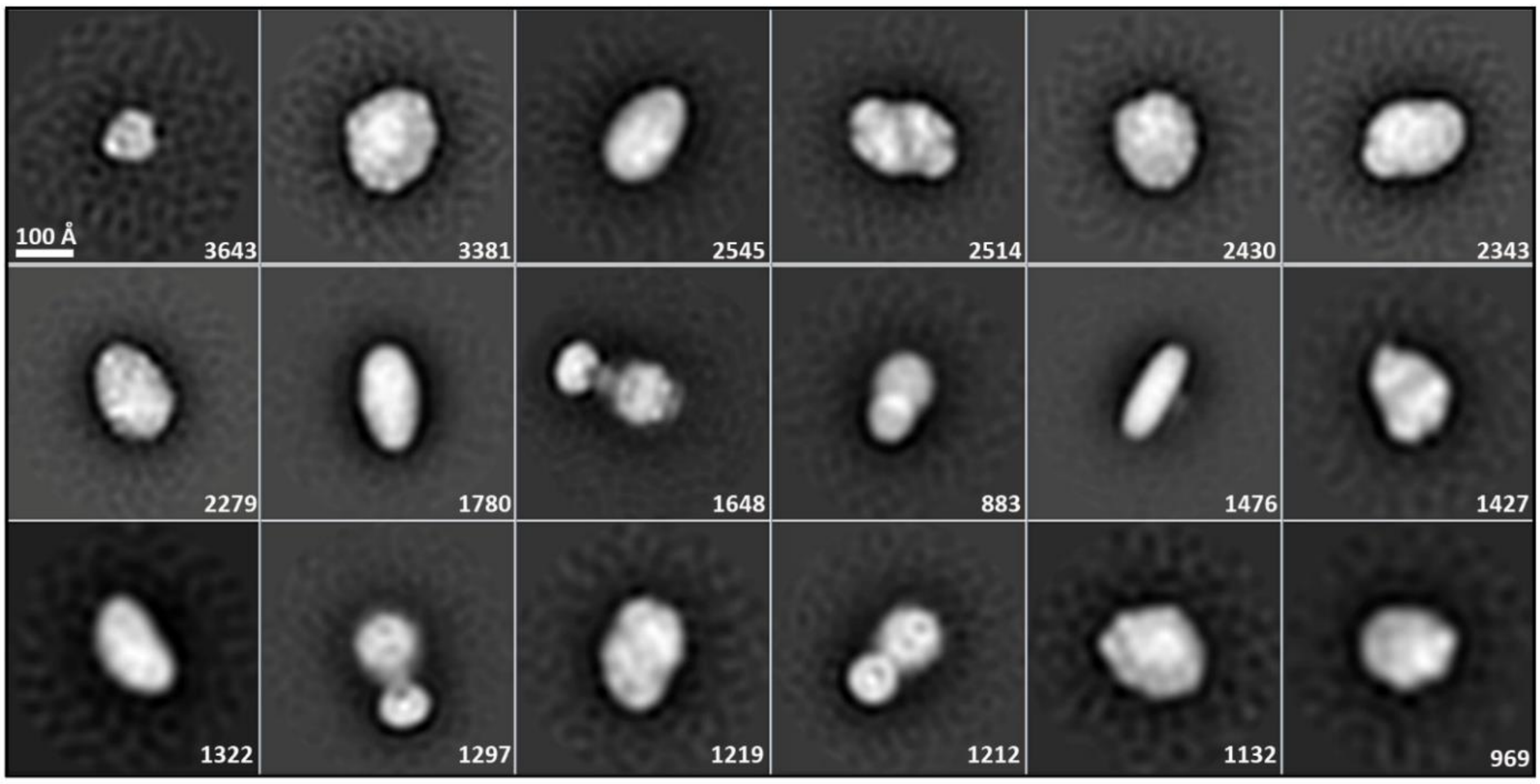

Figure S7 Single-particle analysis and 2D classification of PSI/C_2/CS band from the NoM mutant (see representative gel figure in Figure 5A). The image represents the selection of the most abundant classes. Numbers of averaged projections in given classes are indicated.

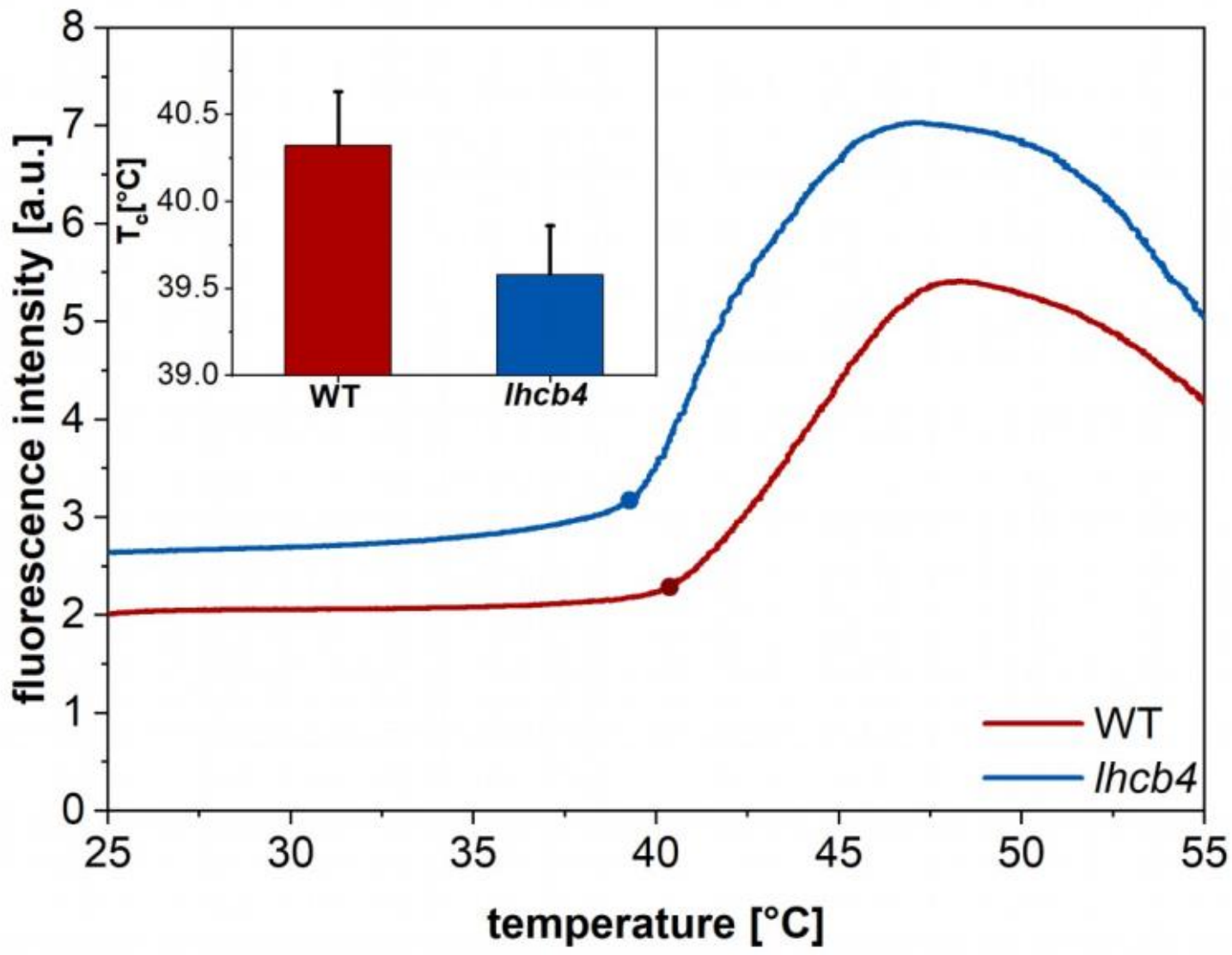

Figure S8 Chlorophyll fluorescence temperature curves in Arabidopsis WT and *lhcb4* mutant. Detached leaves were heated from room temperature to 70°C in a water bath at a rate of 2°C min^{-1}. Simultaneously, chlorophyll fluorescence intensity under weak excitation (F_O level, minimal fluorescence) was recorded (see Ilík et al., 2018). Leaves were dark-adapted for 30 min. The maximum curvature points (T_C) of the initial fluorescence increase are indicated. Insert: The mean of T_C and SD for WT and *lhcb4*. Representative curves are presented. Number of repetitions was 5-6.

Supporting Video

Video S1 Comparison of electron density maps of „canonical“ and „packed“ C_2S_2 supercomplex particles separated by CN-PAGE from thylakoid membranes of the *lhcb4* mutant. Individual C_2S_2 supercomplex electron density maps were taken from Figure 3B. This presentation supports the view that the „packed“ C_2S_2 particle is formed by a shift of CS parts in the canonical C_2S_2 supercomplex lacking LHCB4.
